# Supplementary material for: Using Google Health Trends to investigate COVID-19 incidence in Africa
Source: PLoS One. 2022 Jun 7;17(6):e0269573. doi: 10.1371/journal.pone.0269573 (PMC9173636; doi:10.1371/journal.pone.0269573)

**Title:** Using Google Health Trends to investigate COVID-19 incidence in Africa

**Authors:** Alexander Fulk, Daniel Romero-Alvarez, Qays Abu-Saymeh, Jarron M. Saint Onge, A. Townsend Peterson, Folashade B. Agusto

**Description:** **Supplementary Figures** - Plots depicting the best and worst performing countries from the weighted regression analysis and plots depicting the multiple linear regression models between COVID-19 case counts and Google Health Trends search queries for the 54 African countries studied.
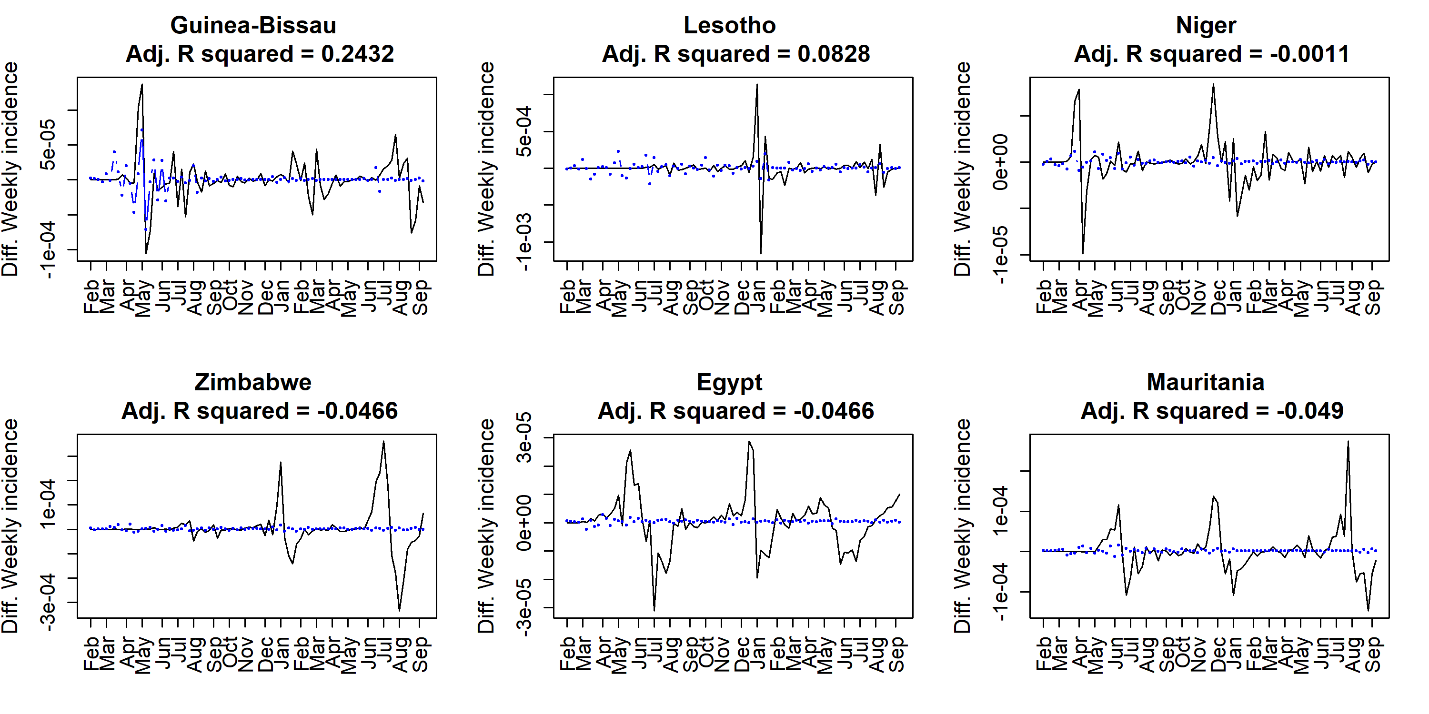


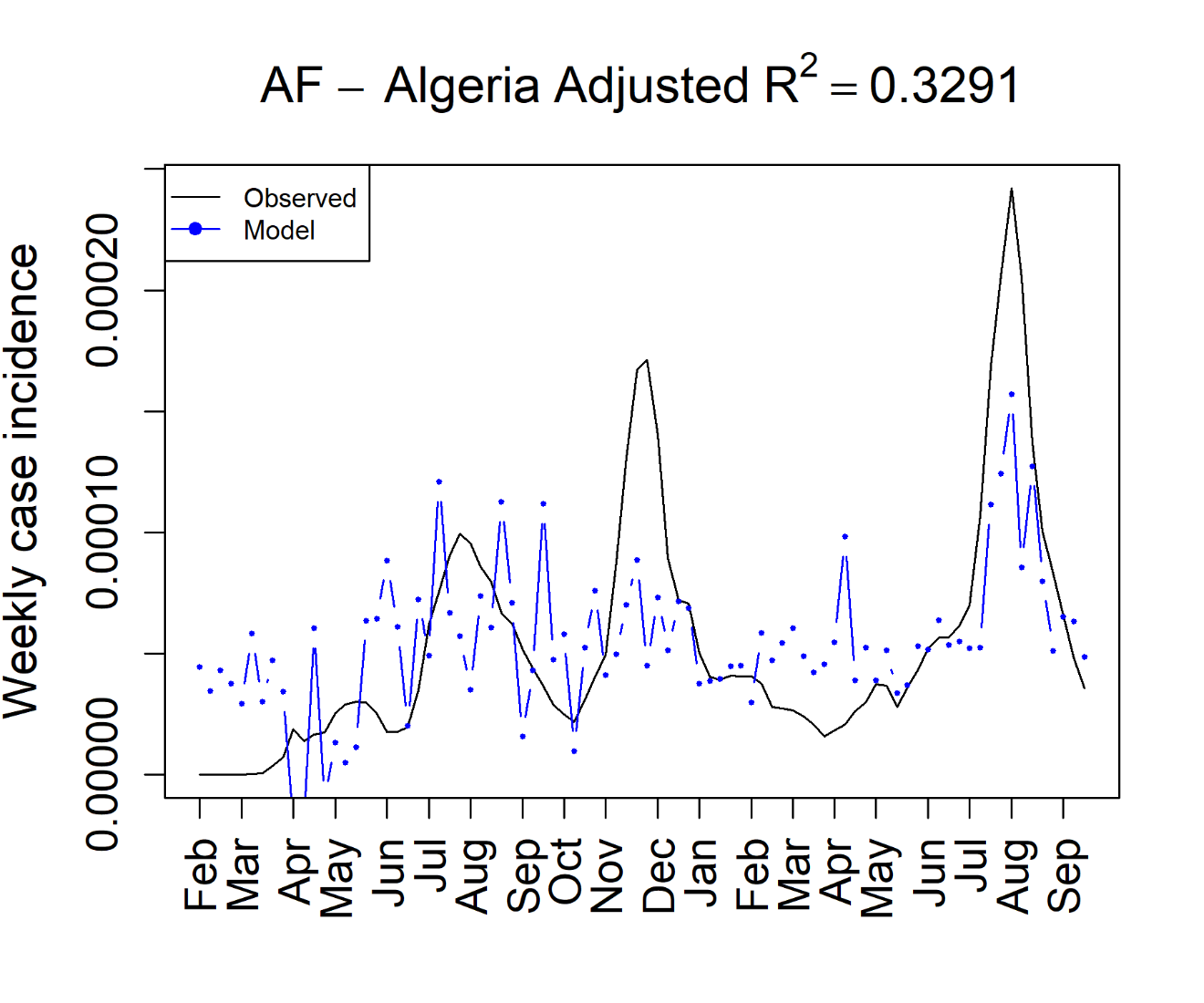

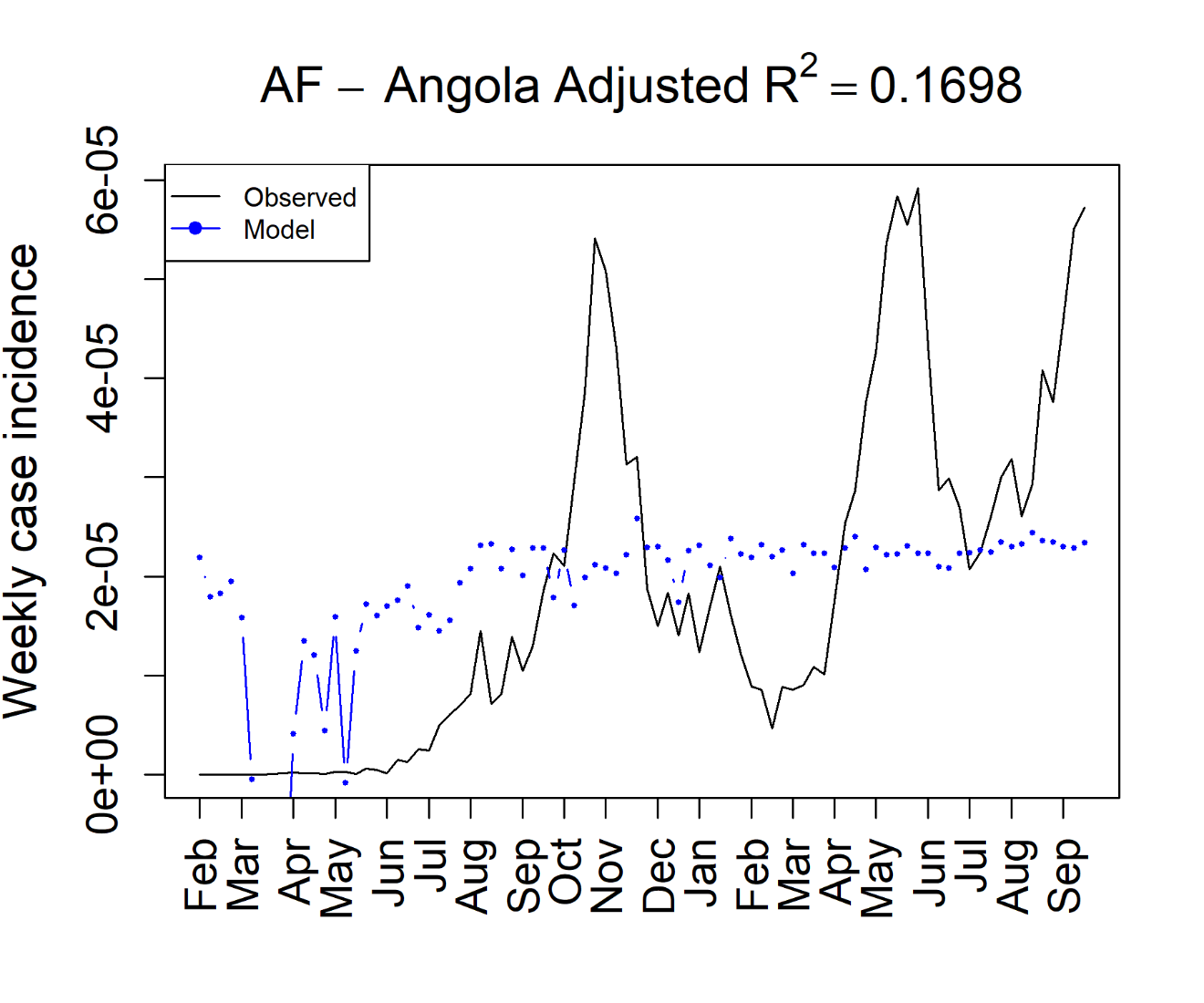

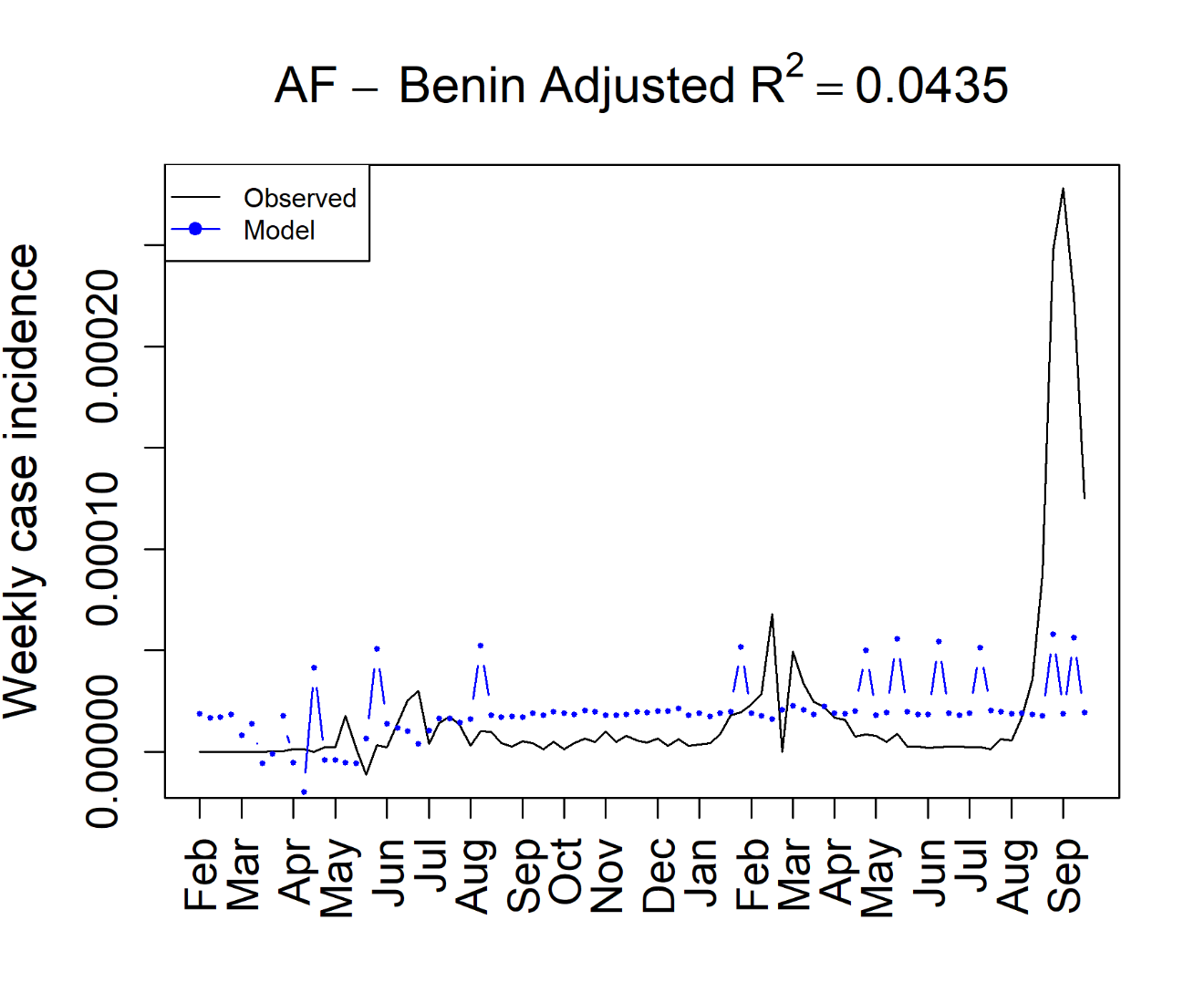

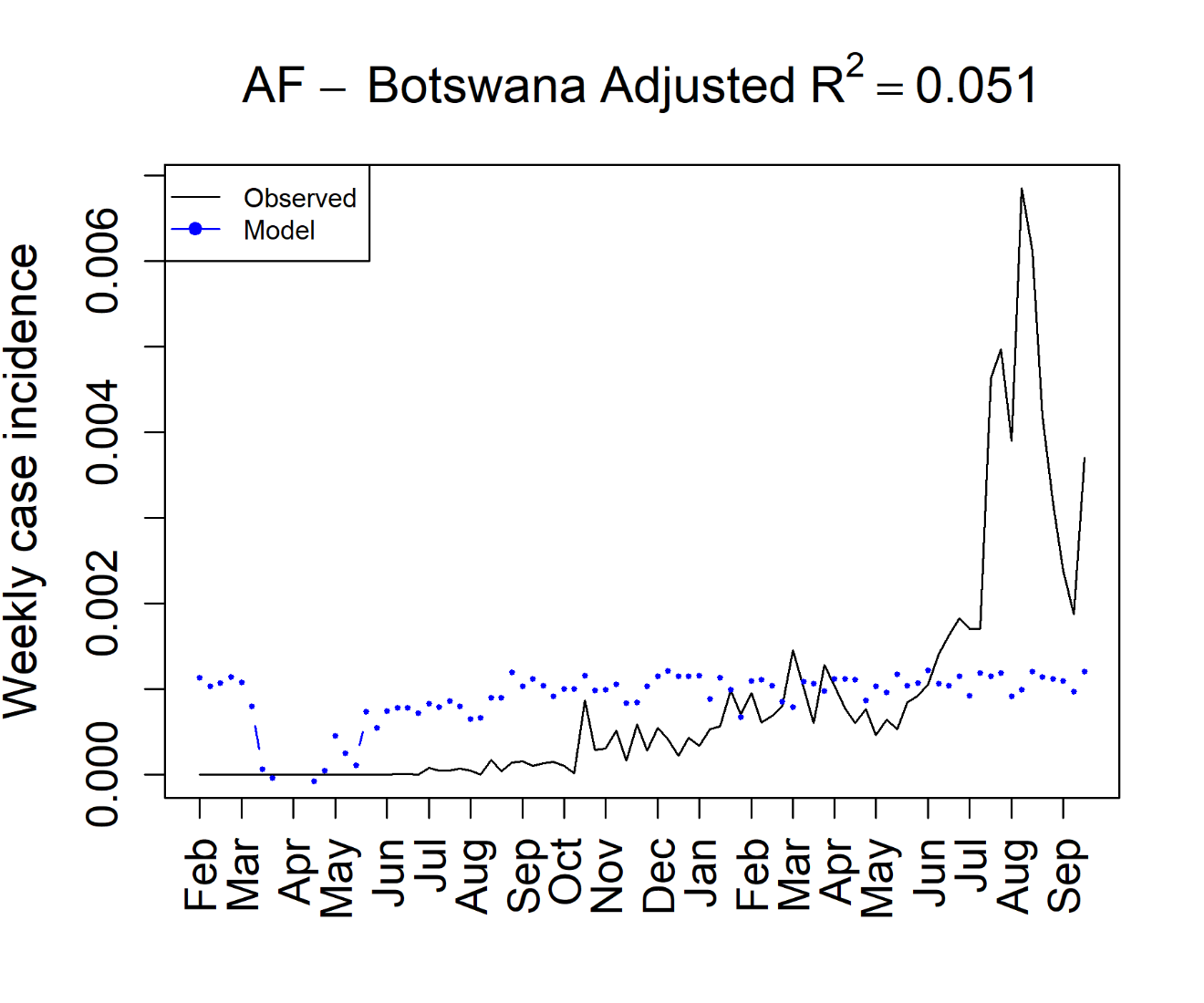

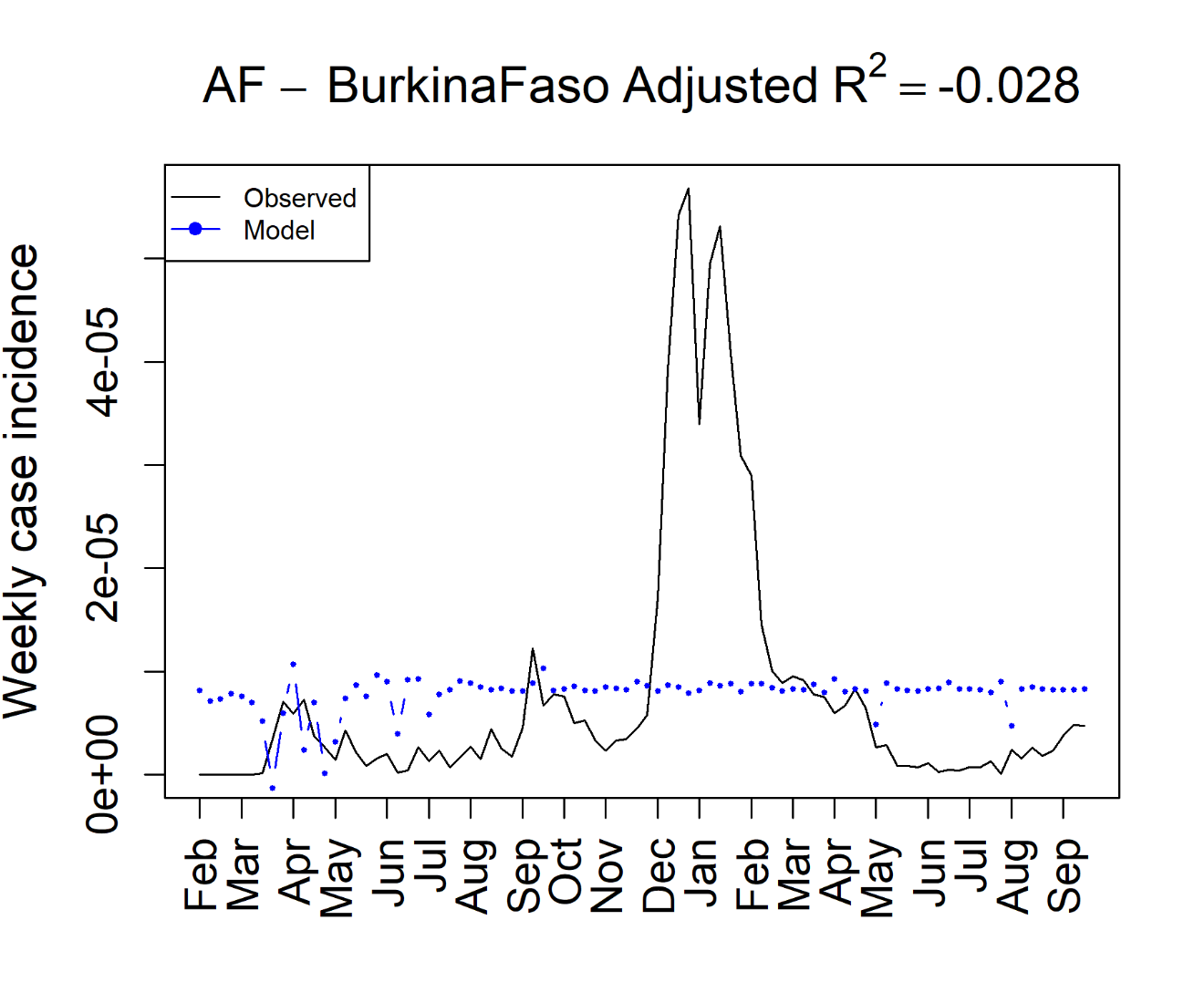

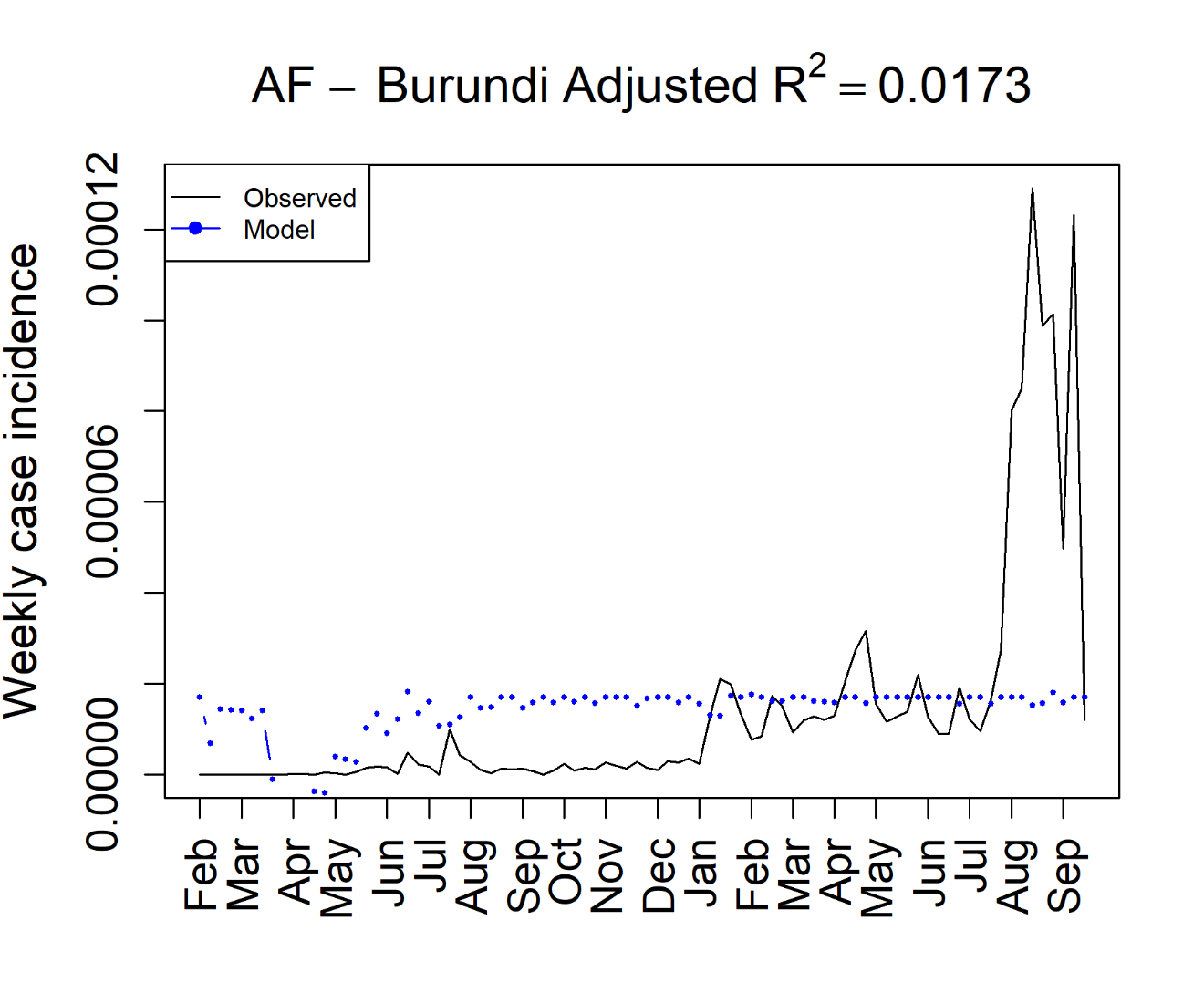

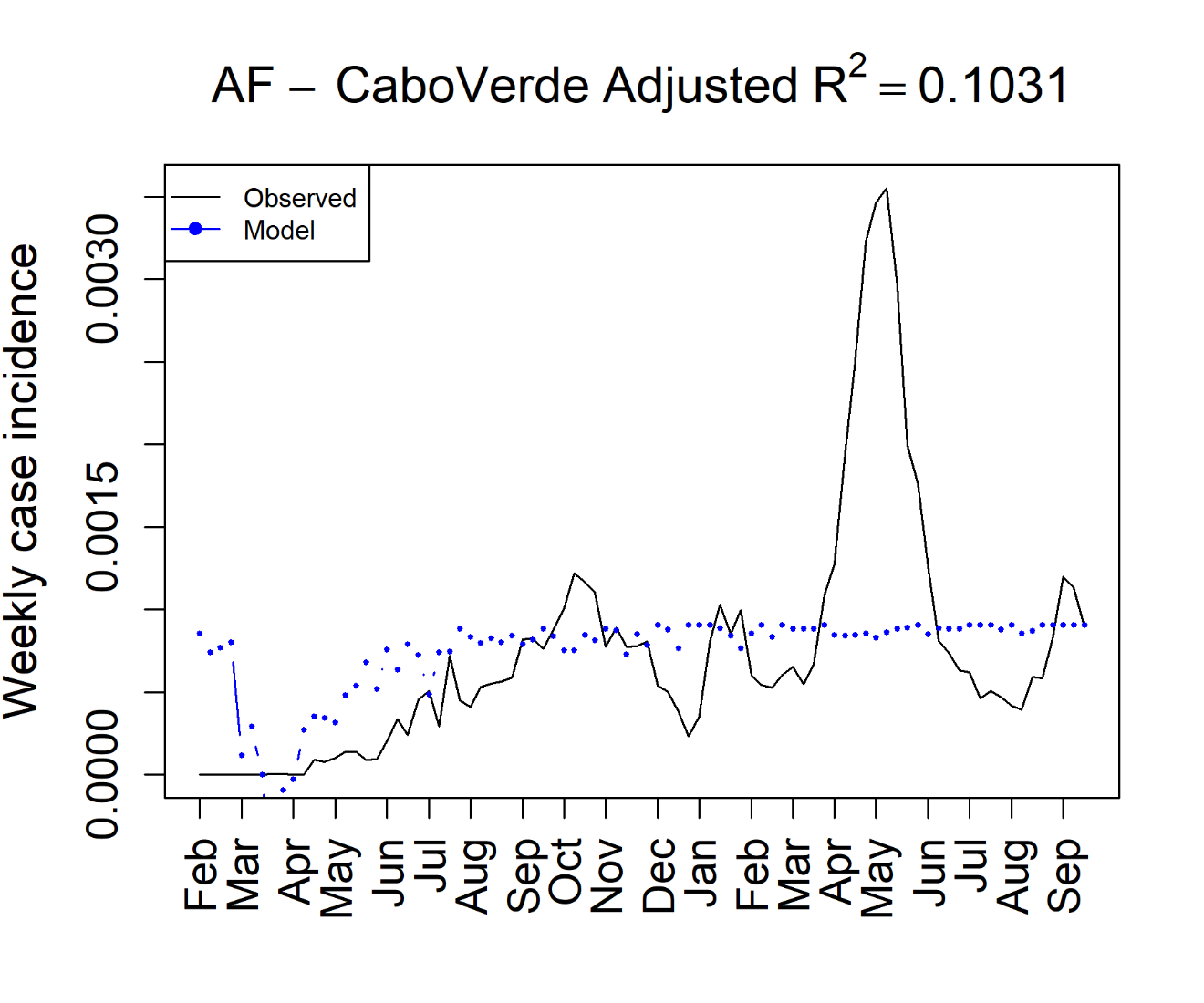

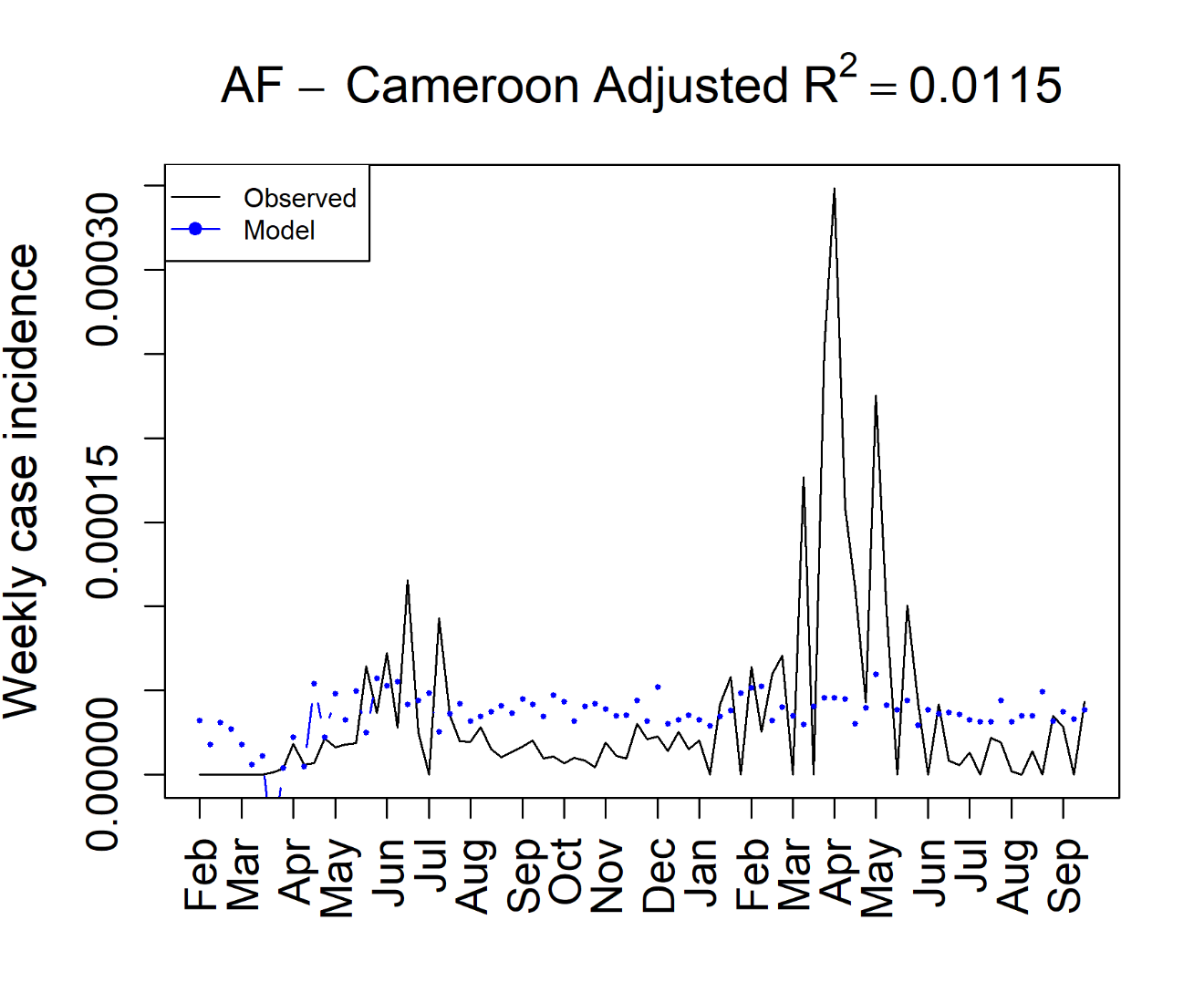

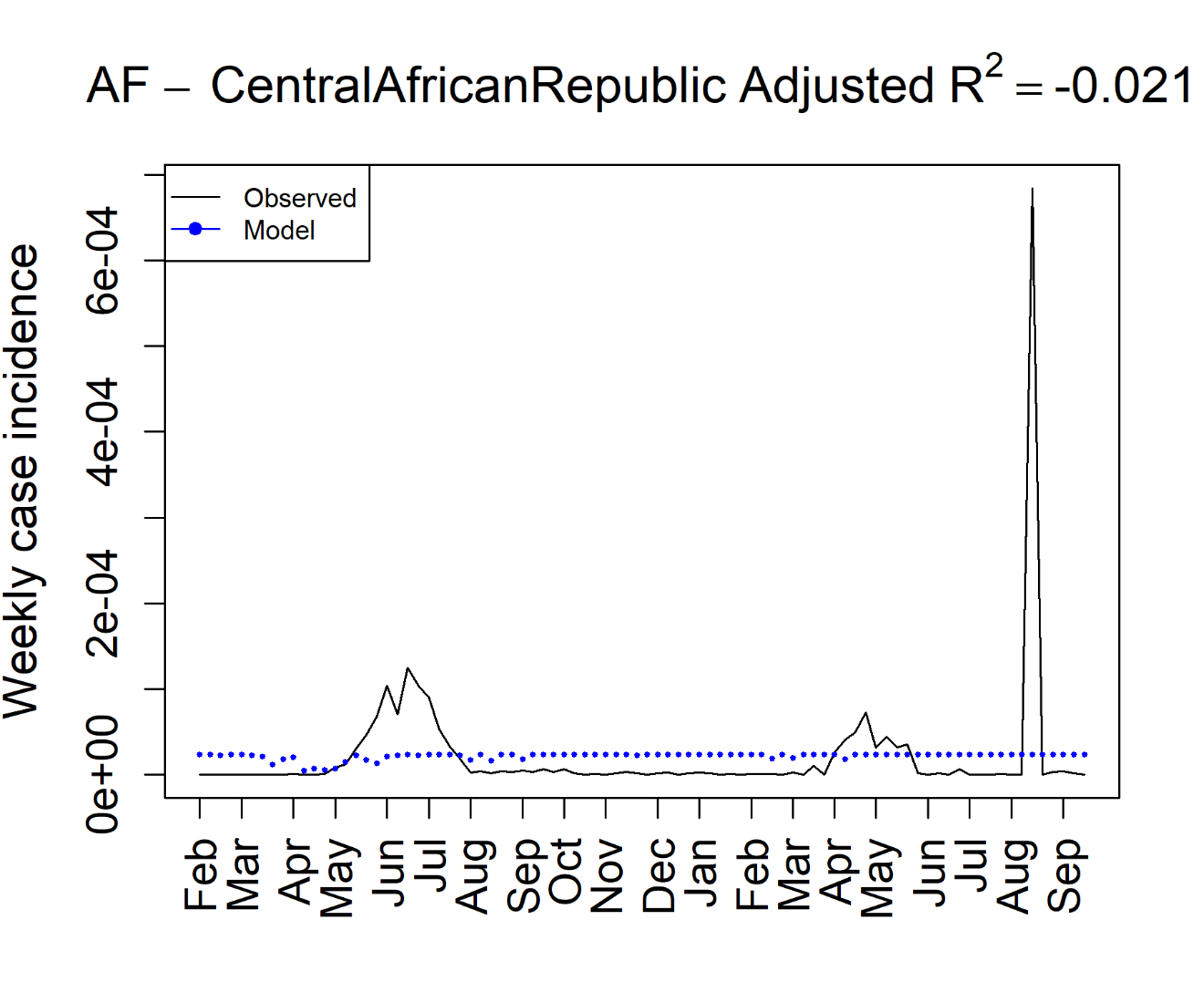

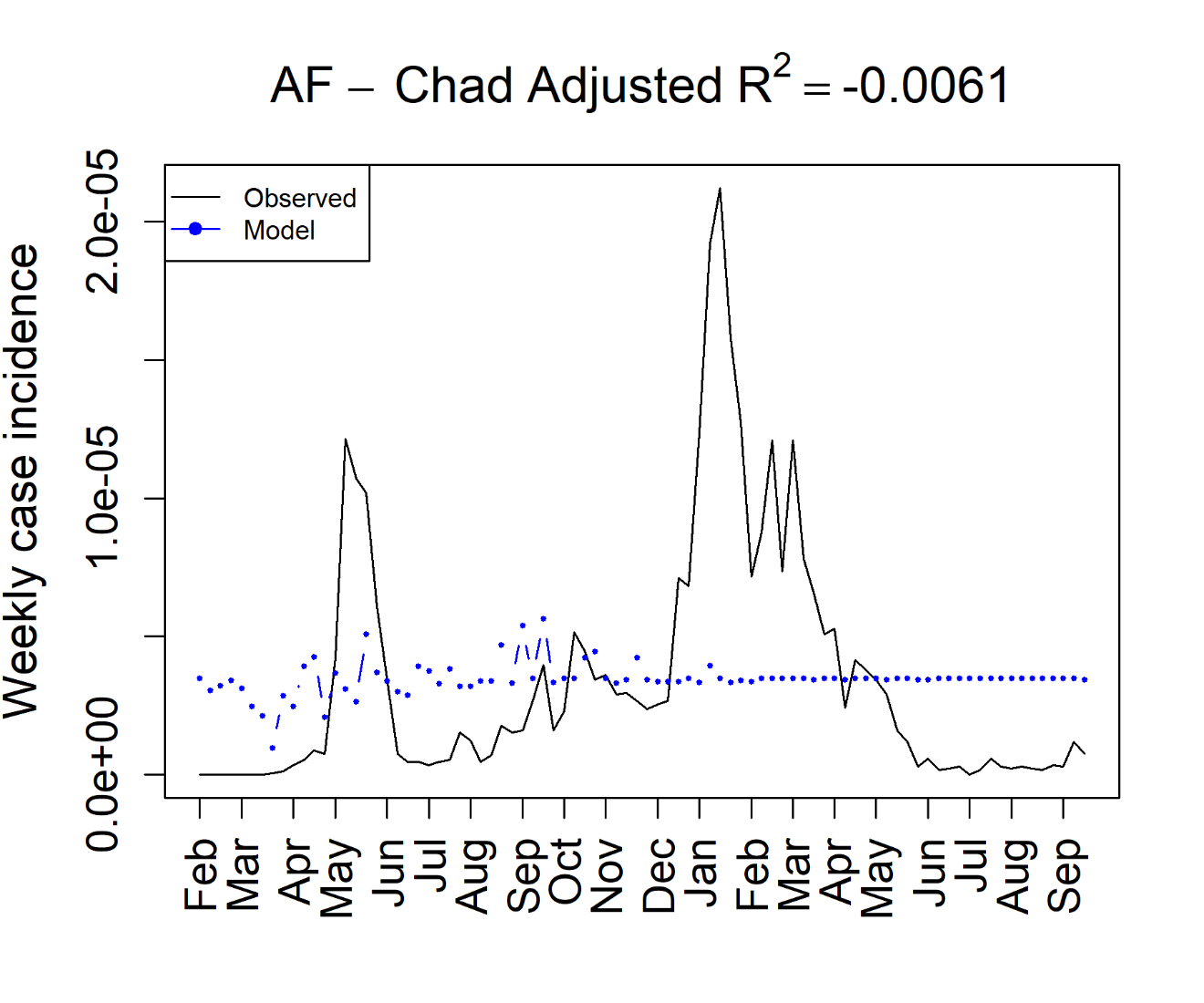

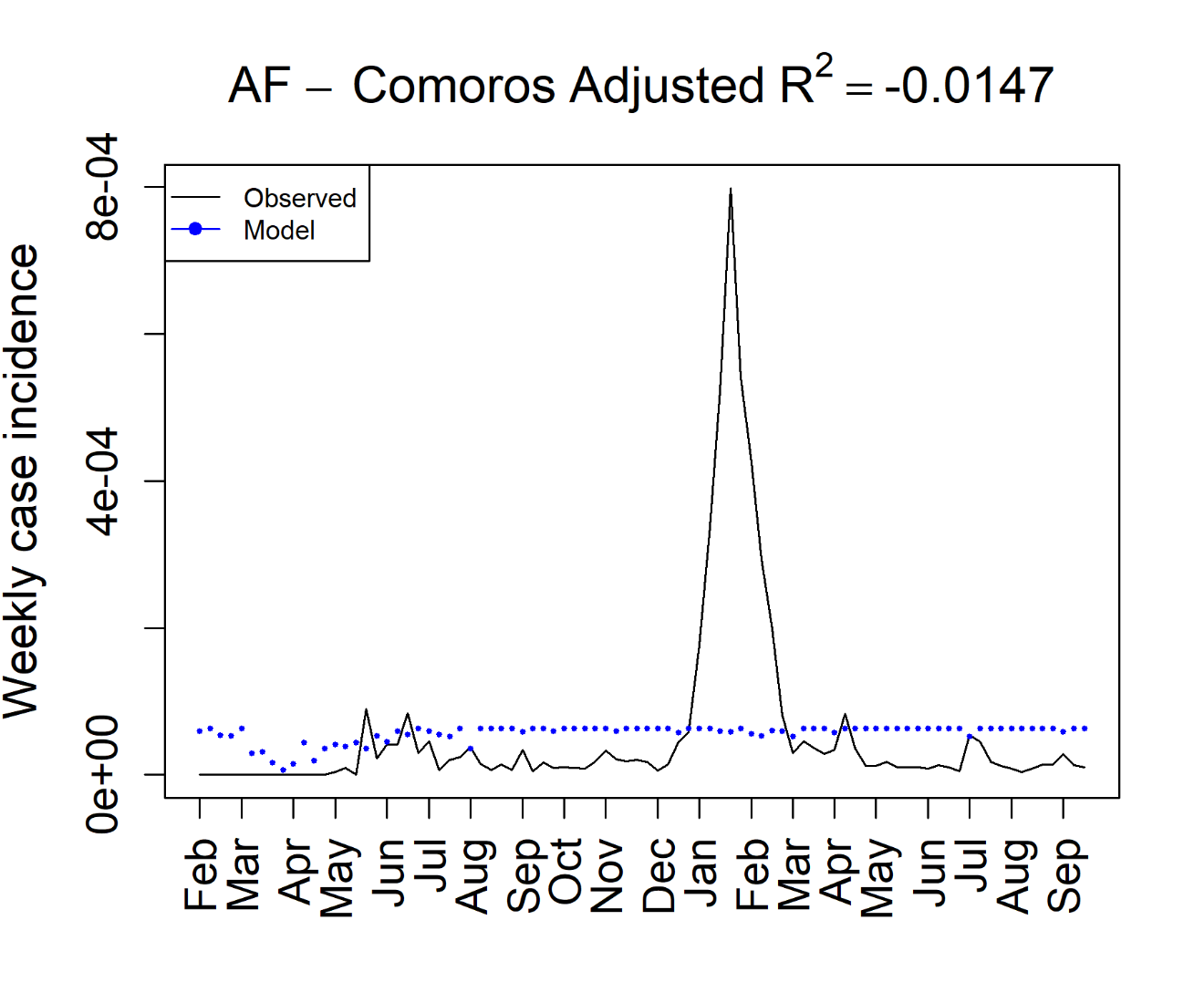

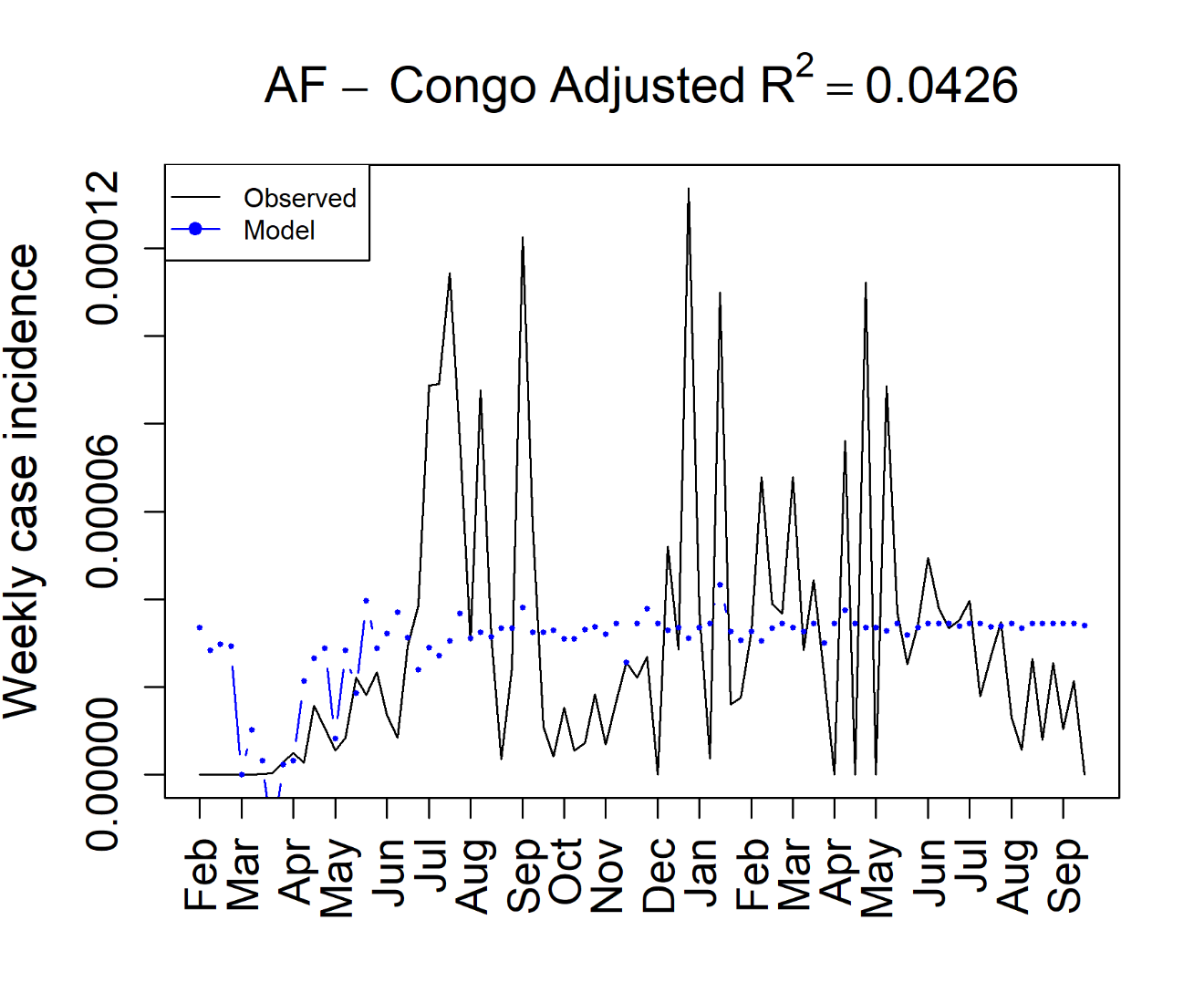

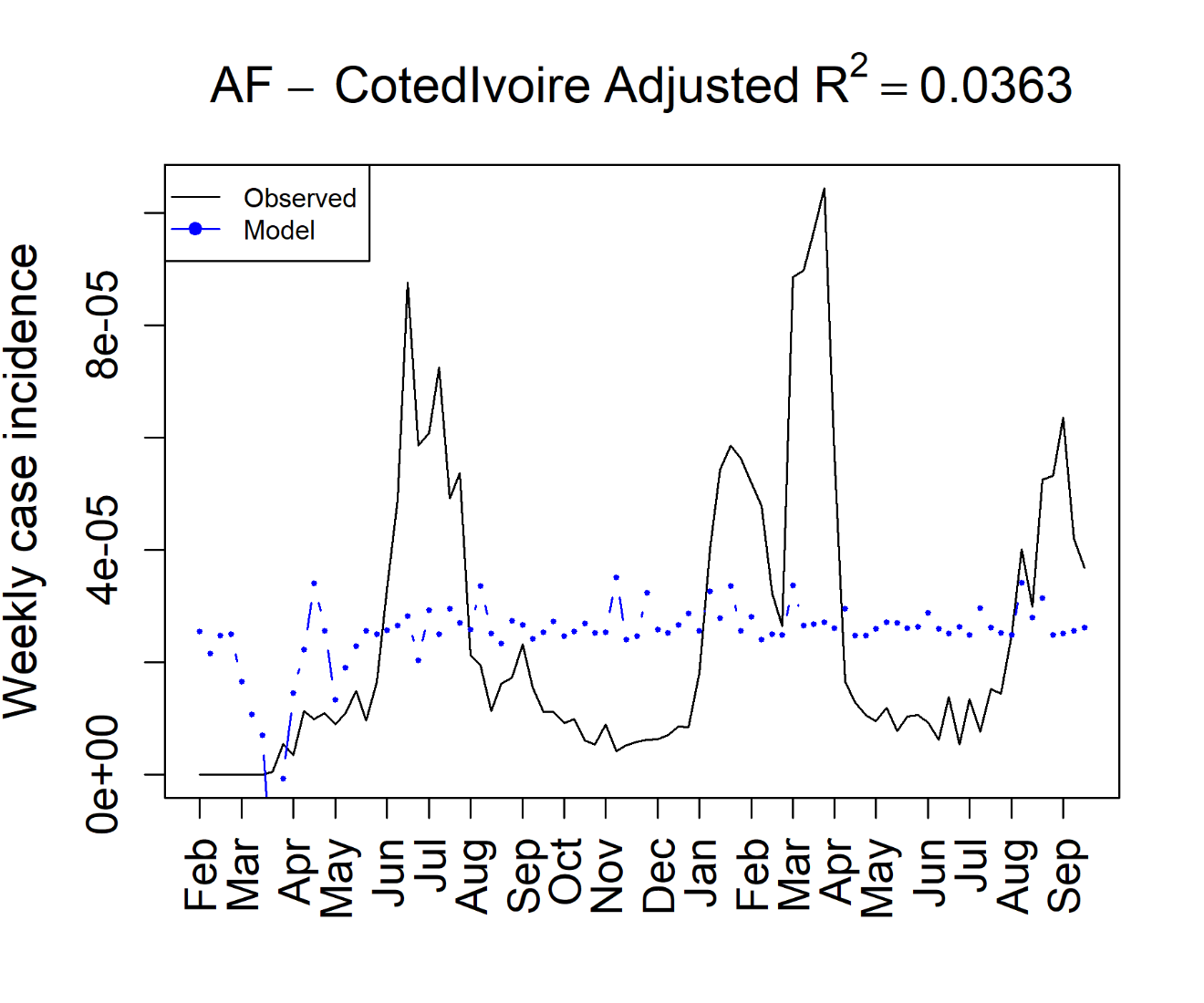

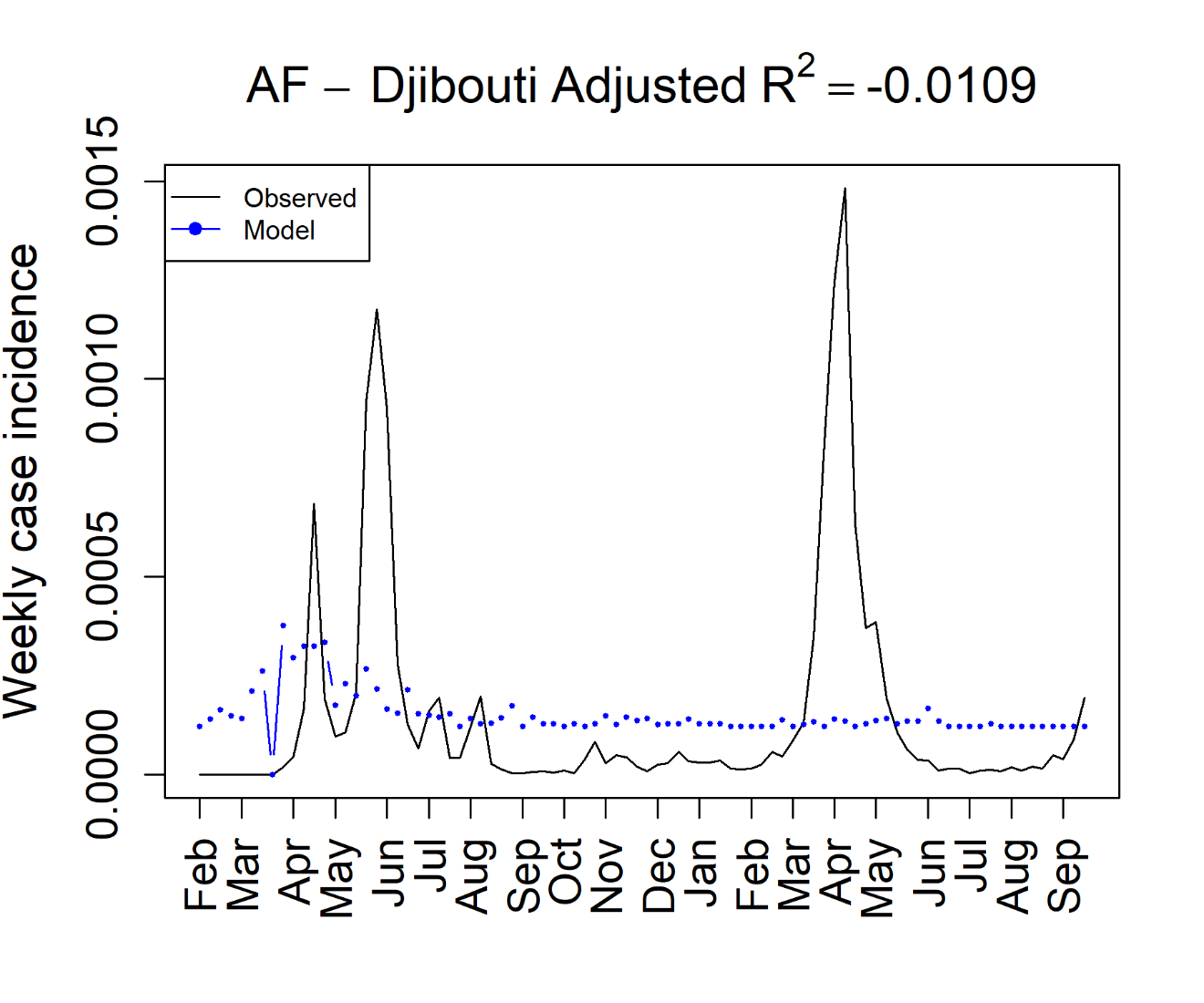

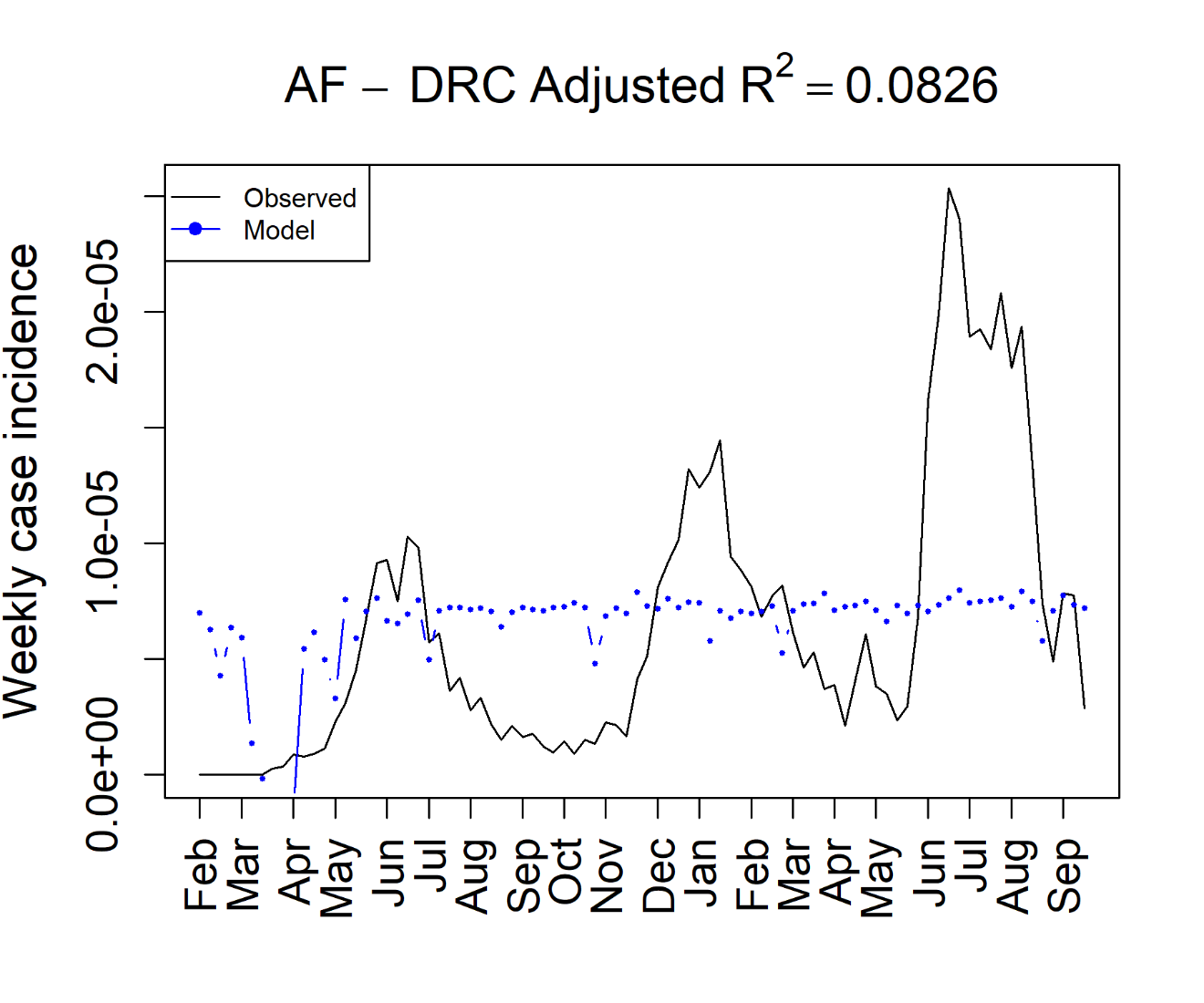

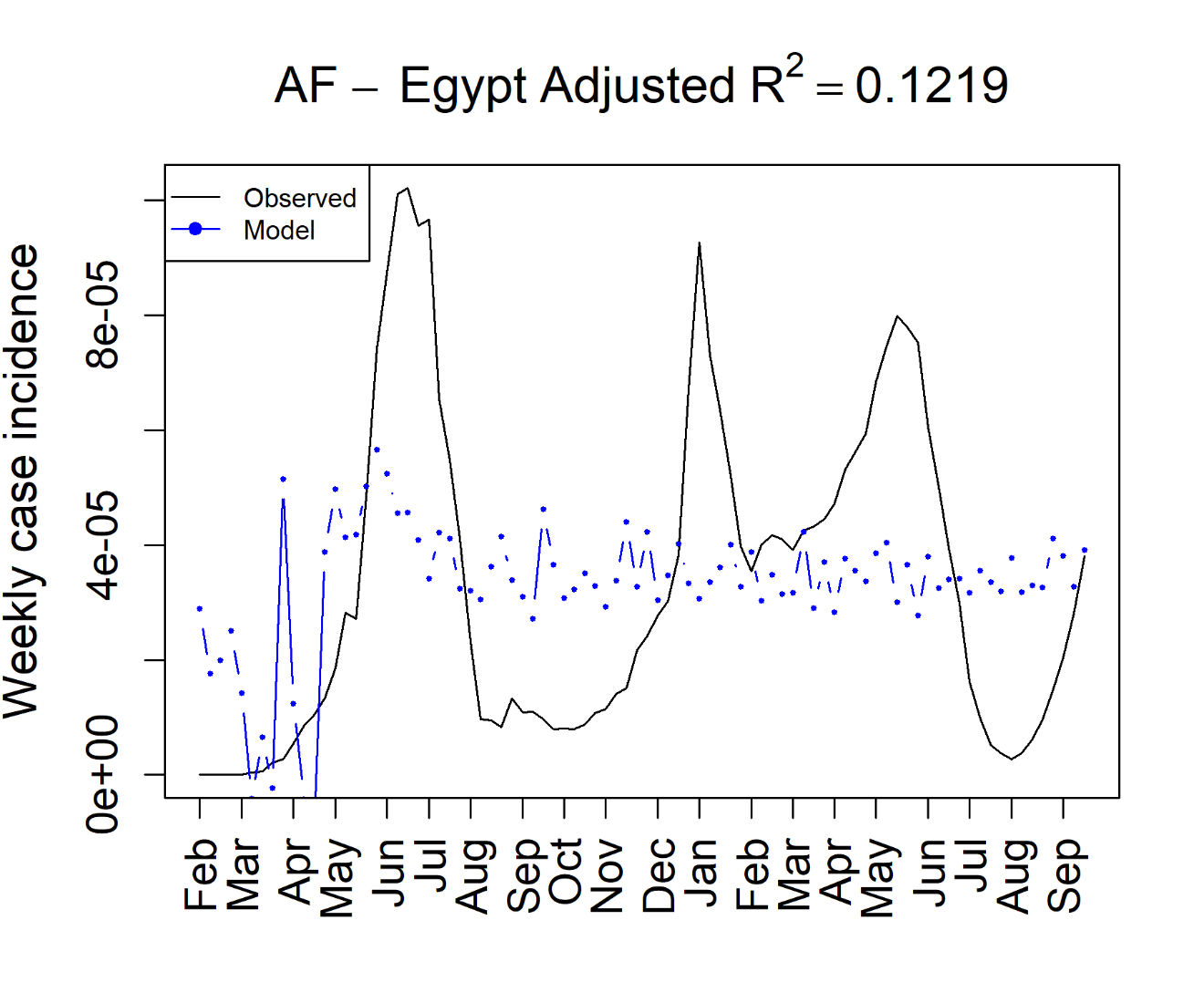

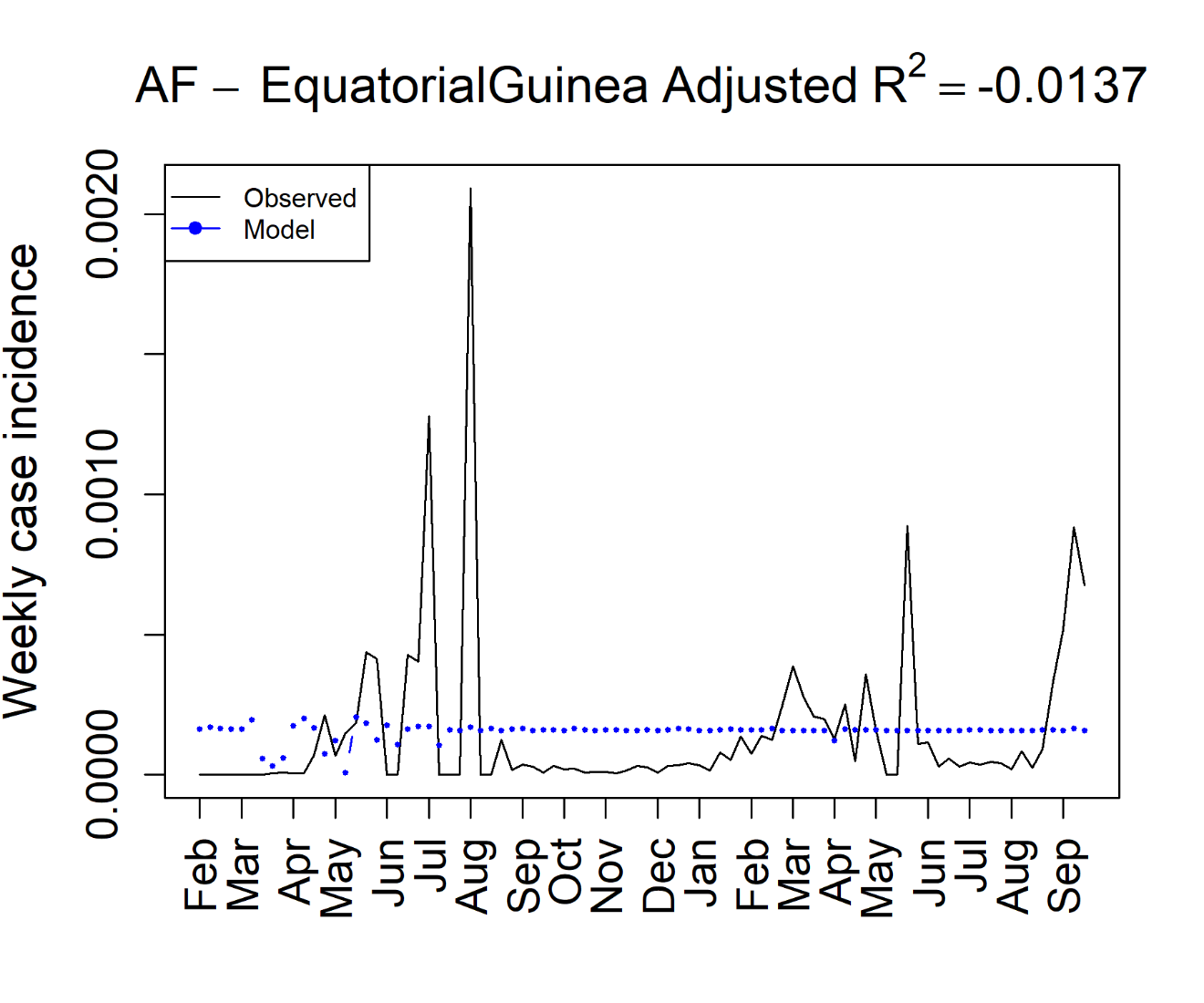

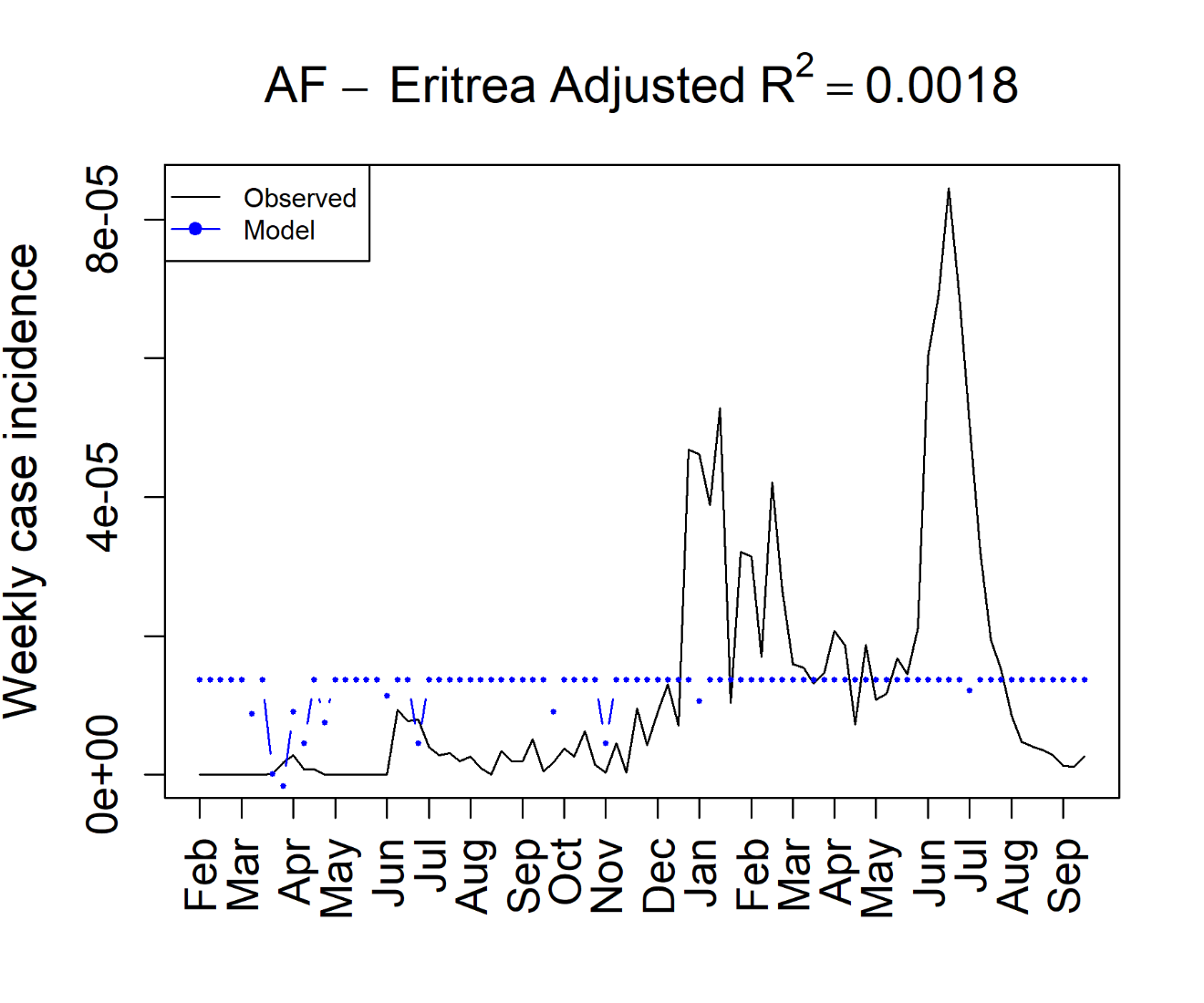

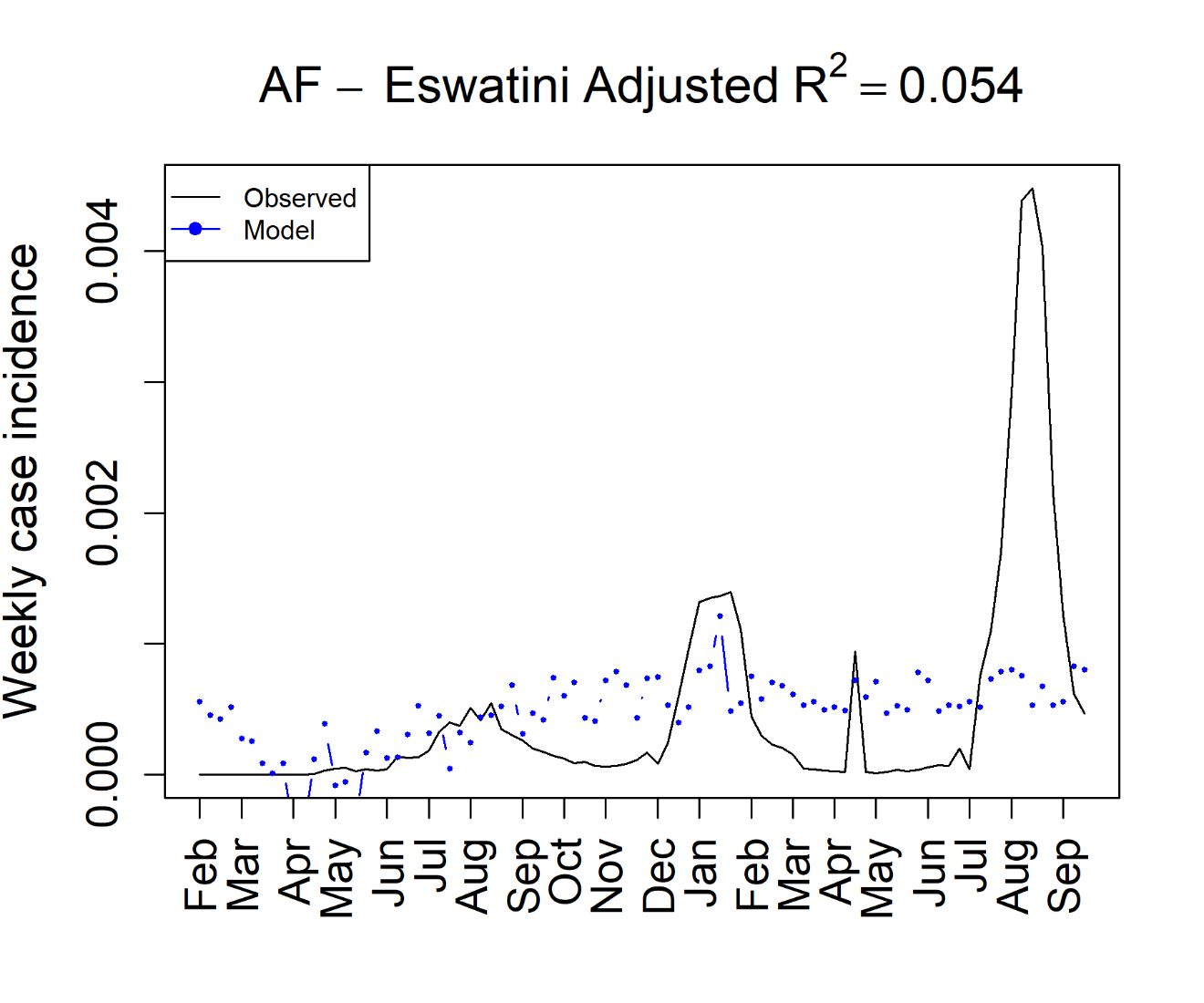

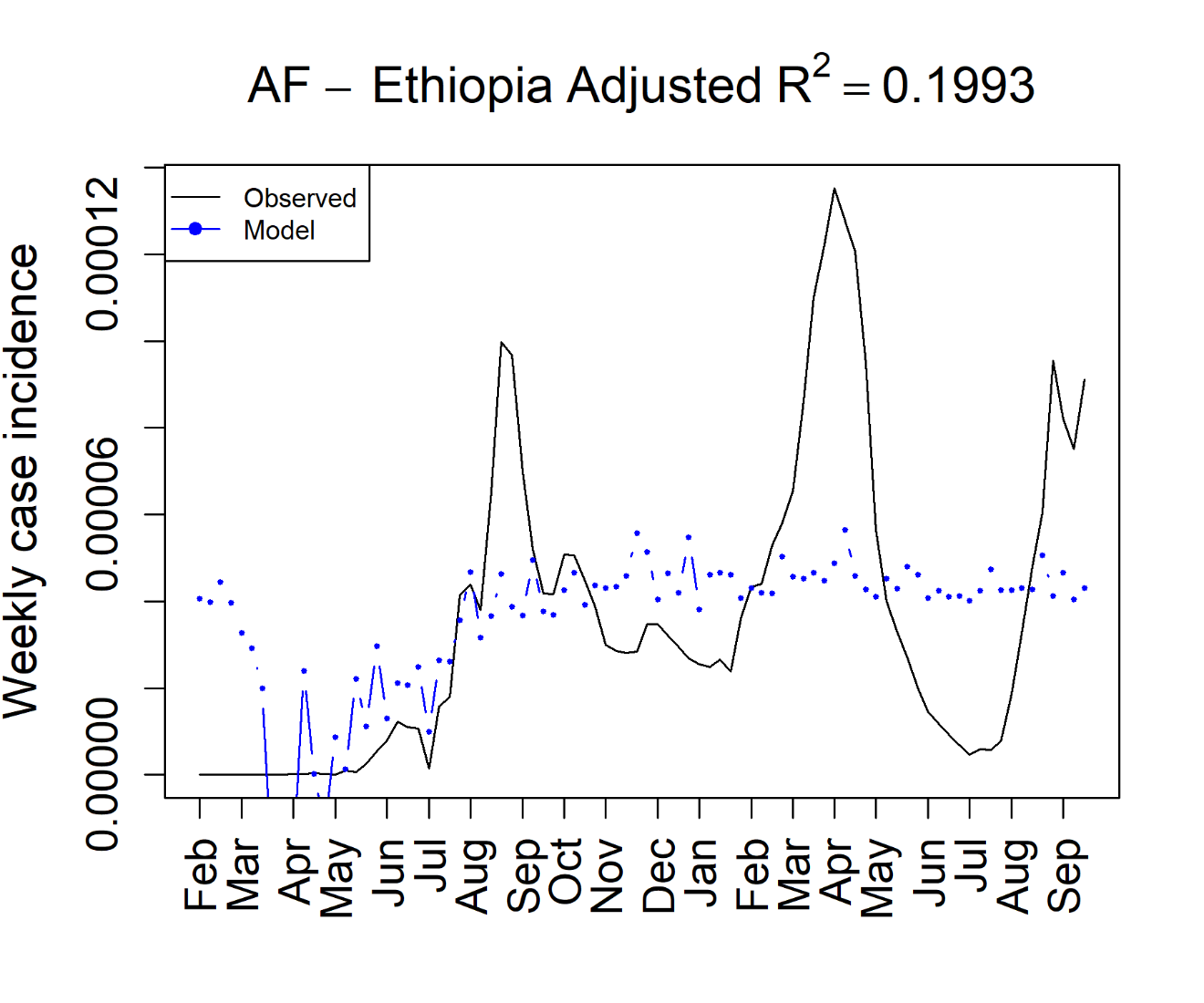

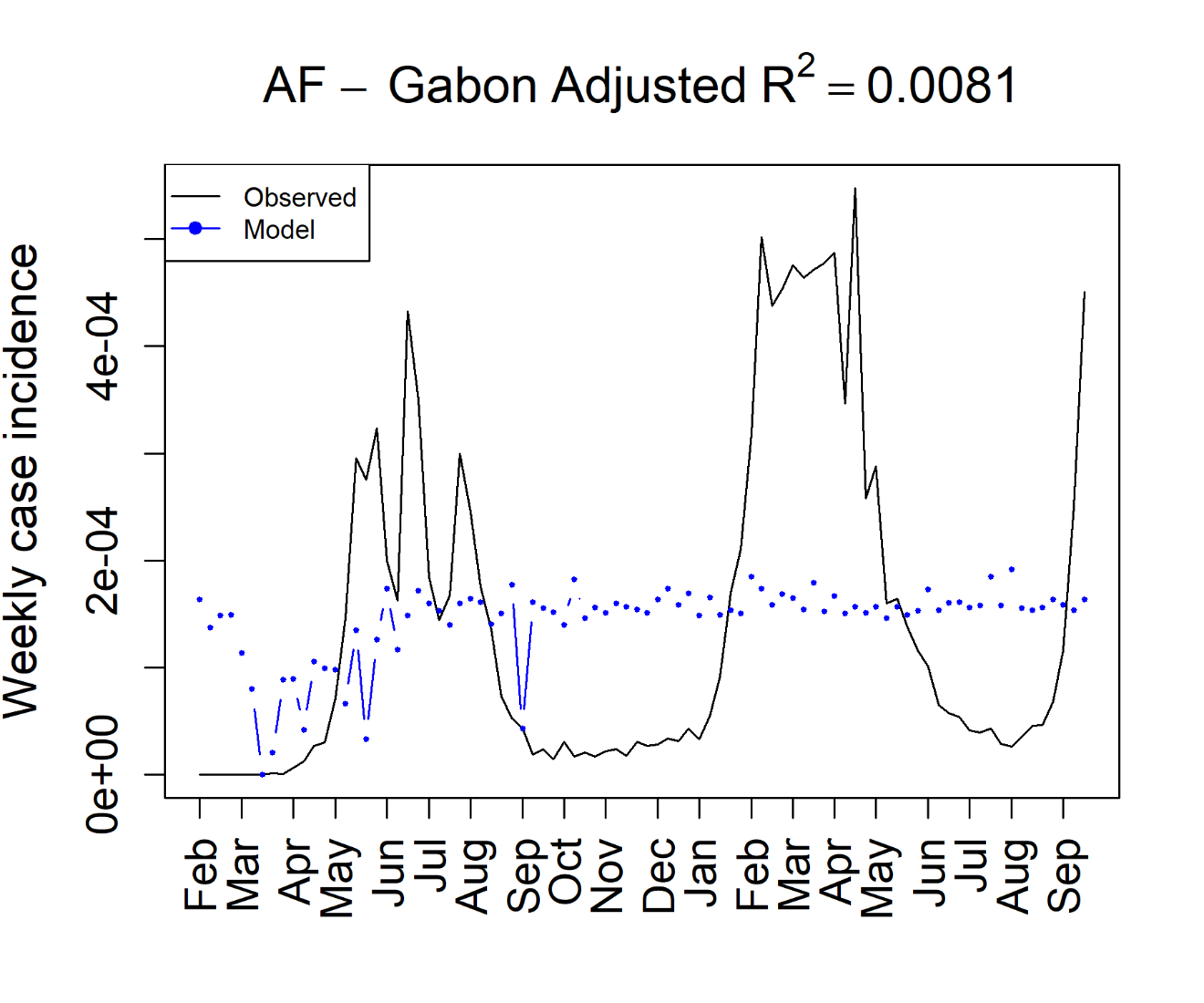

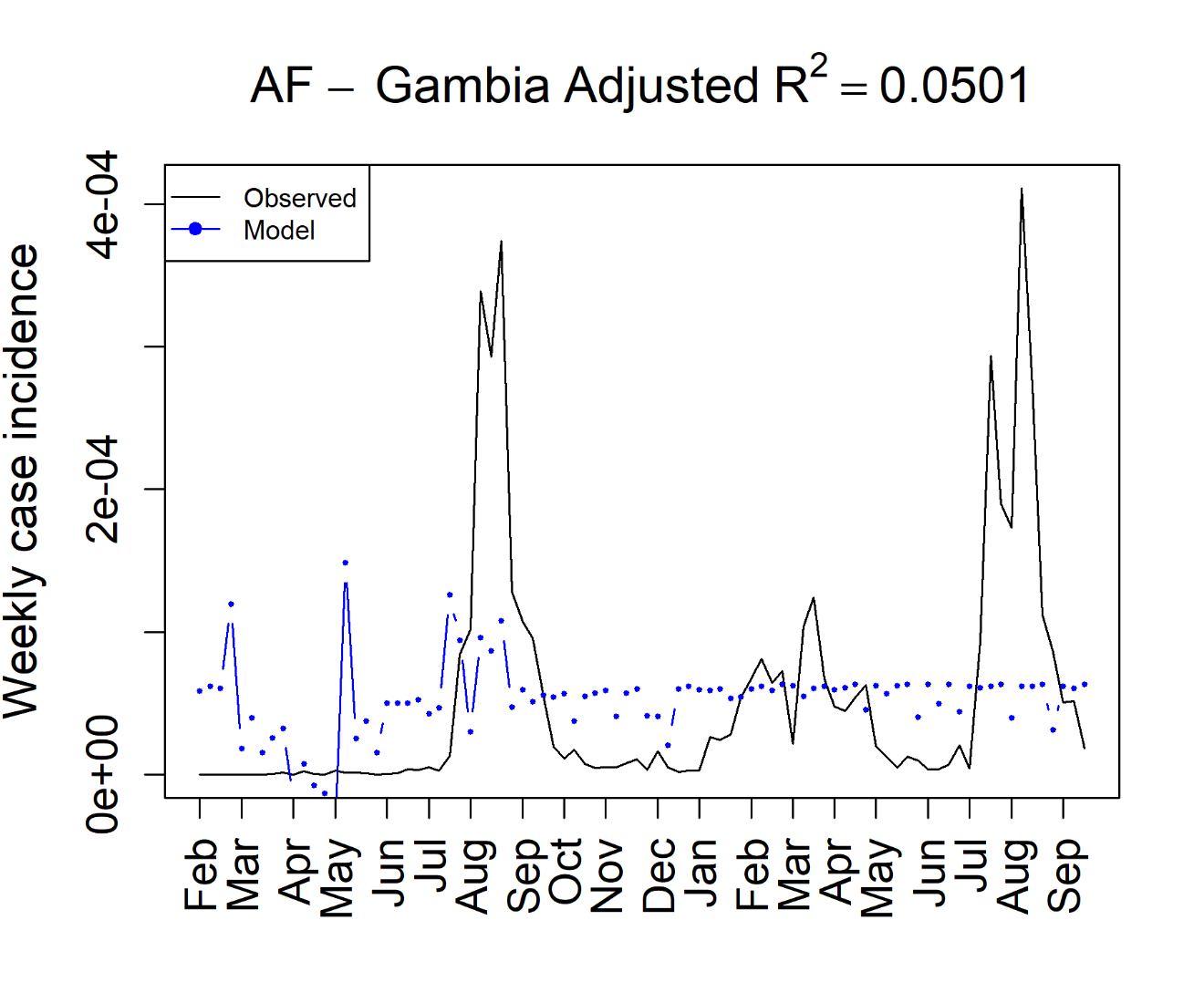

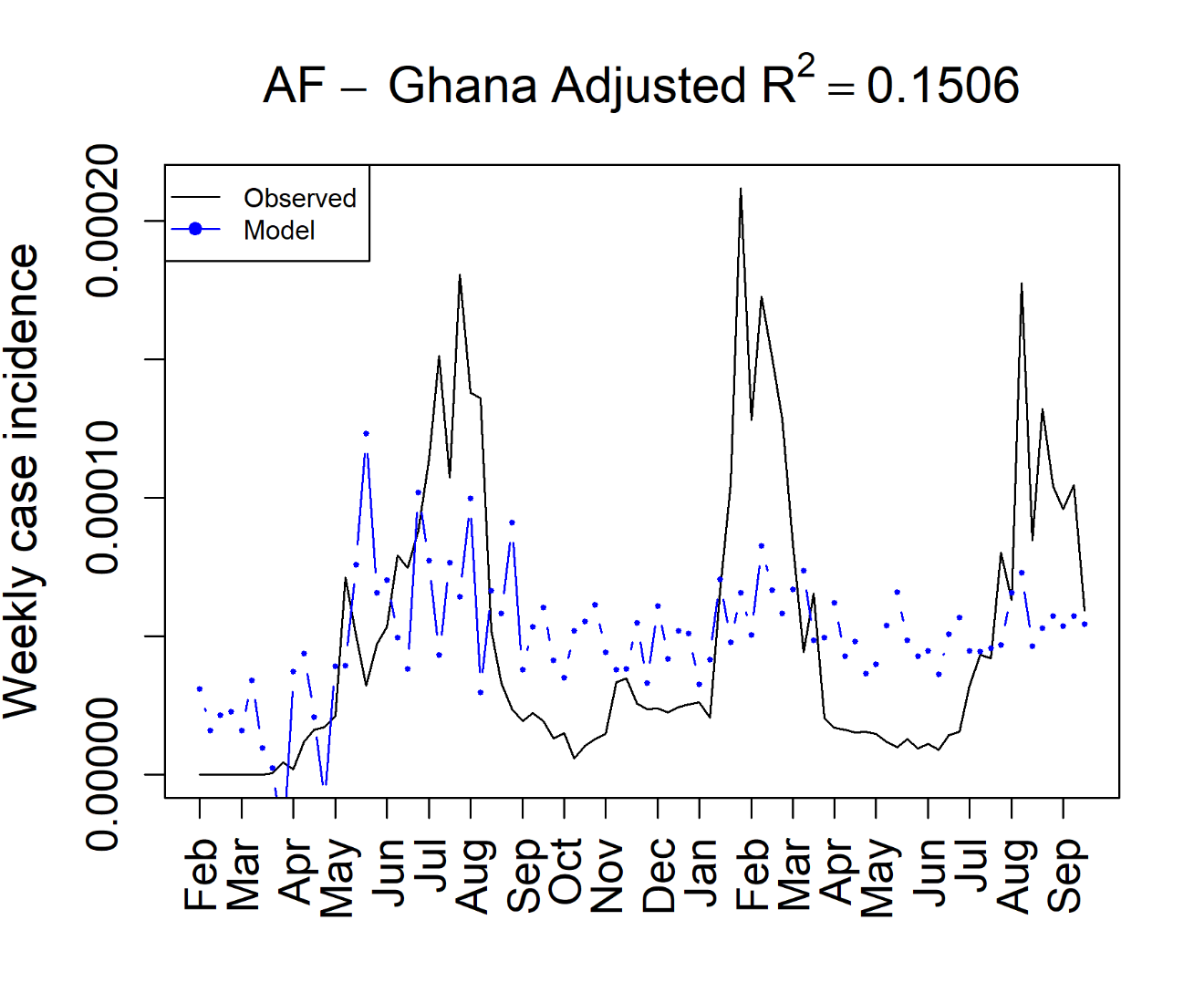

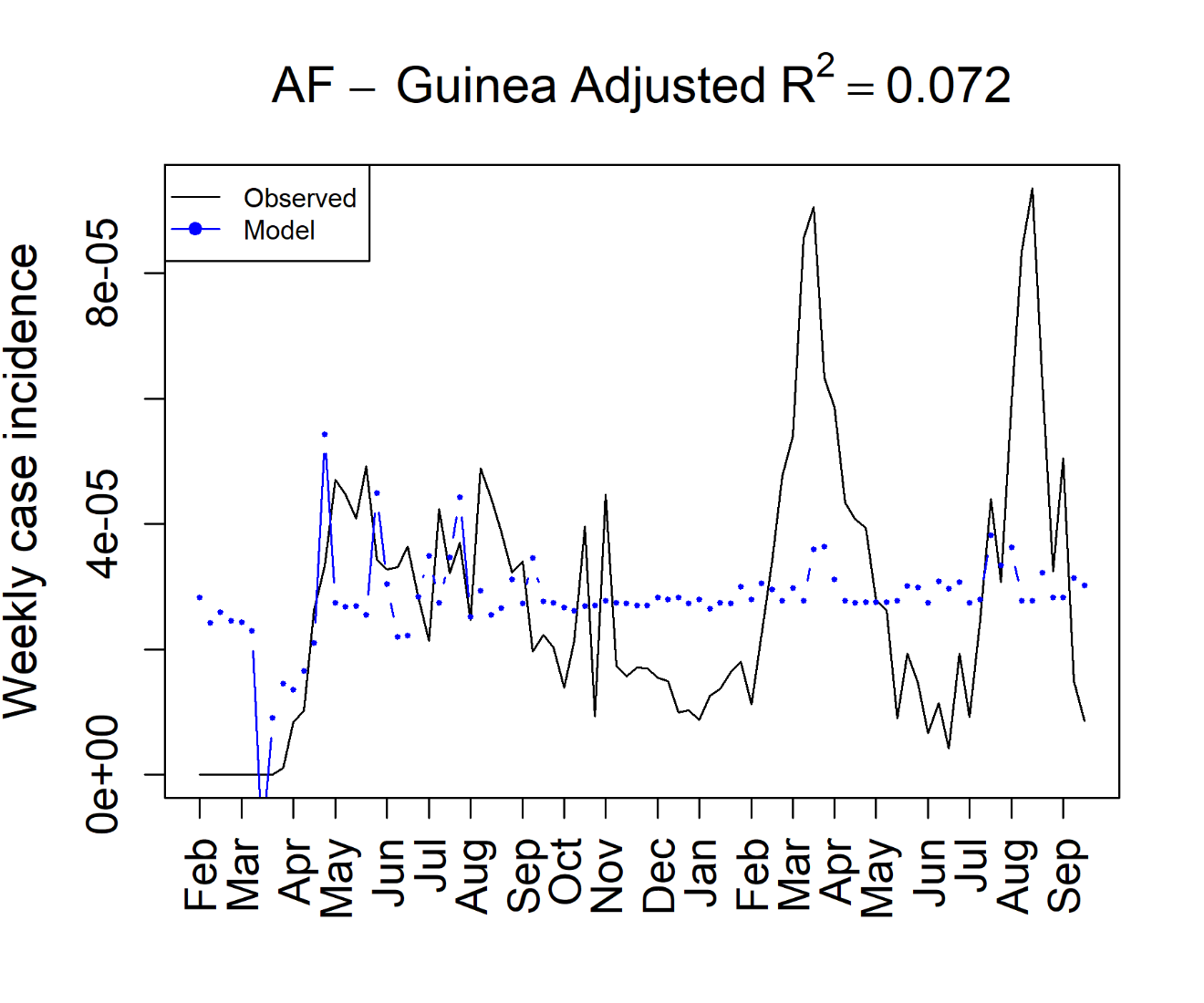

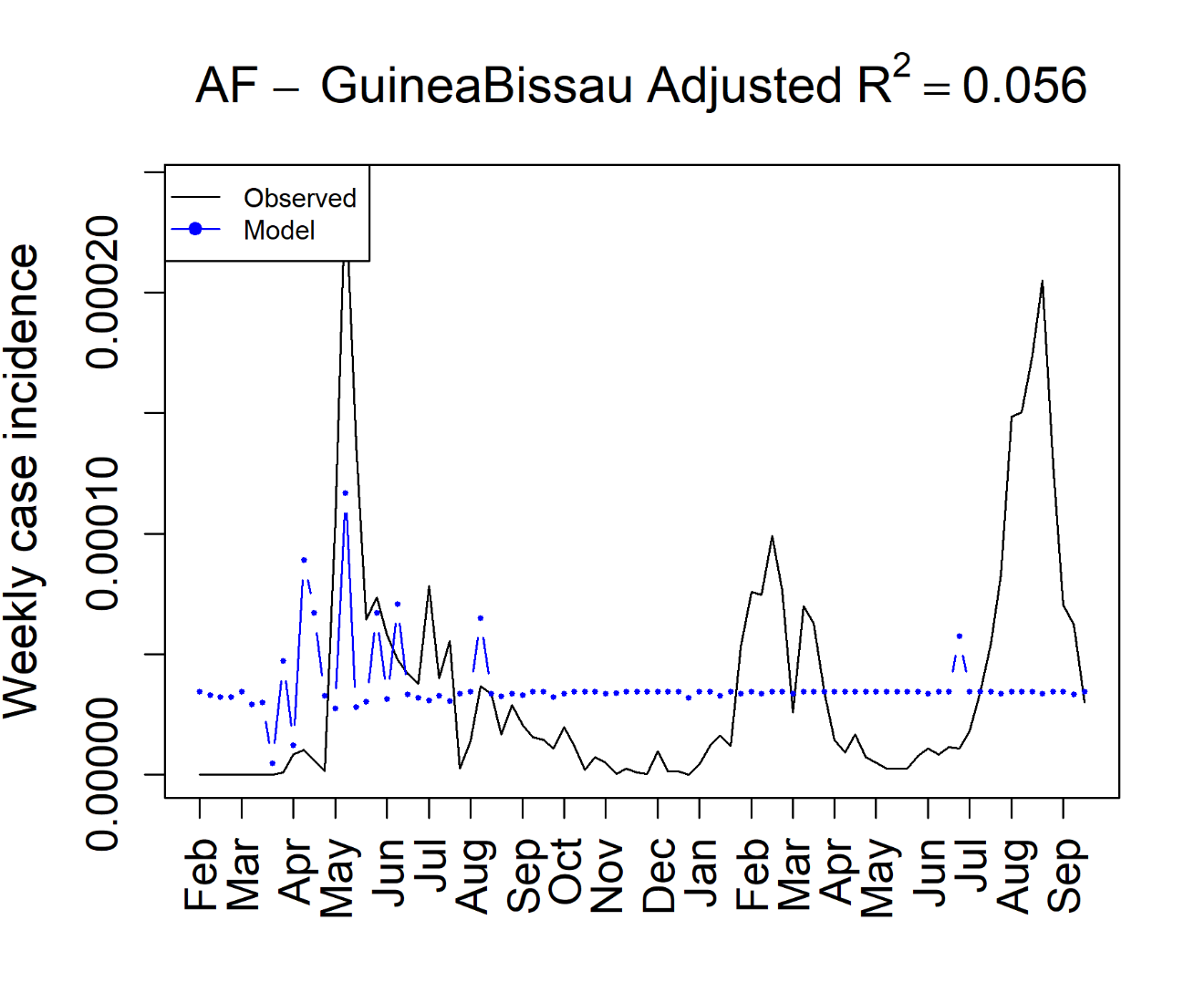

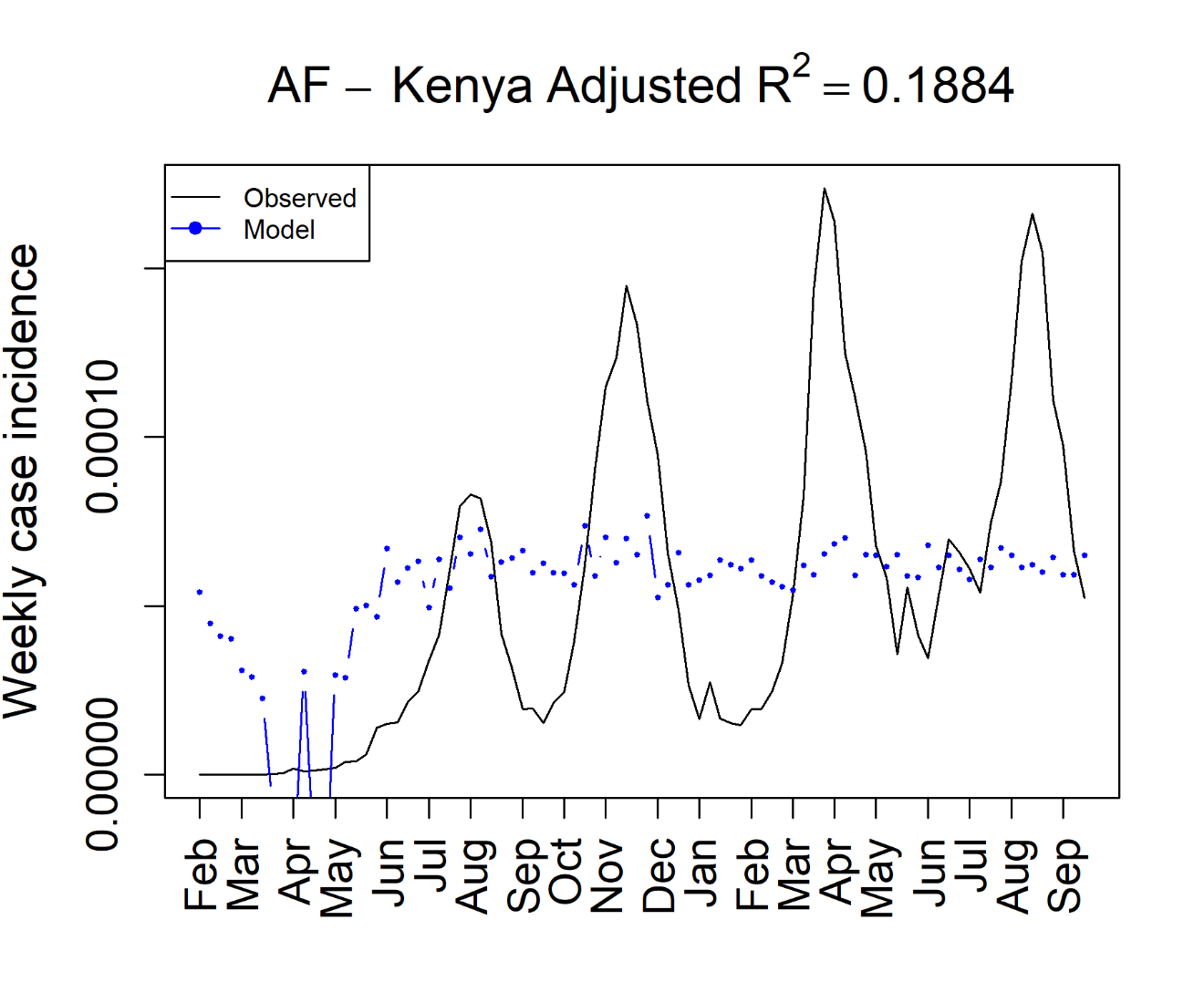

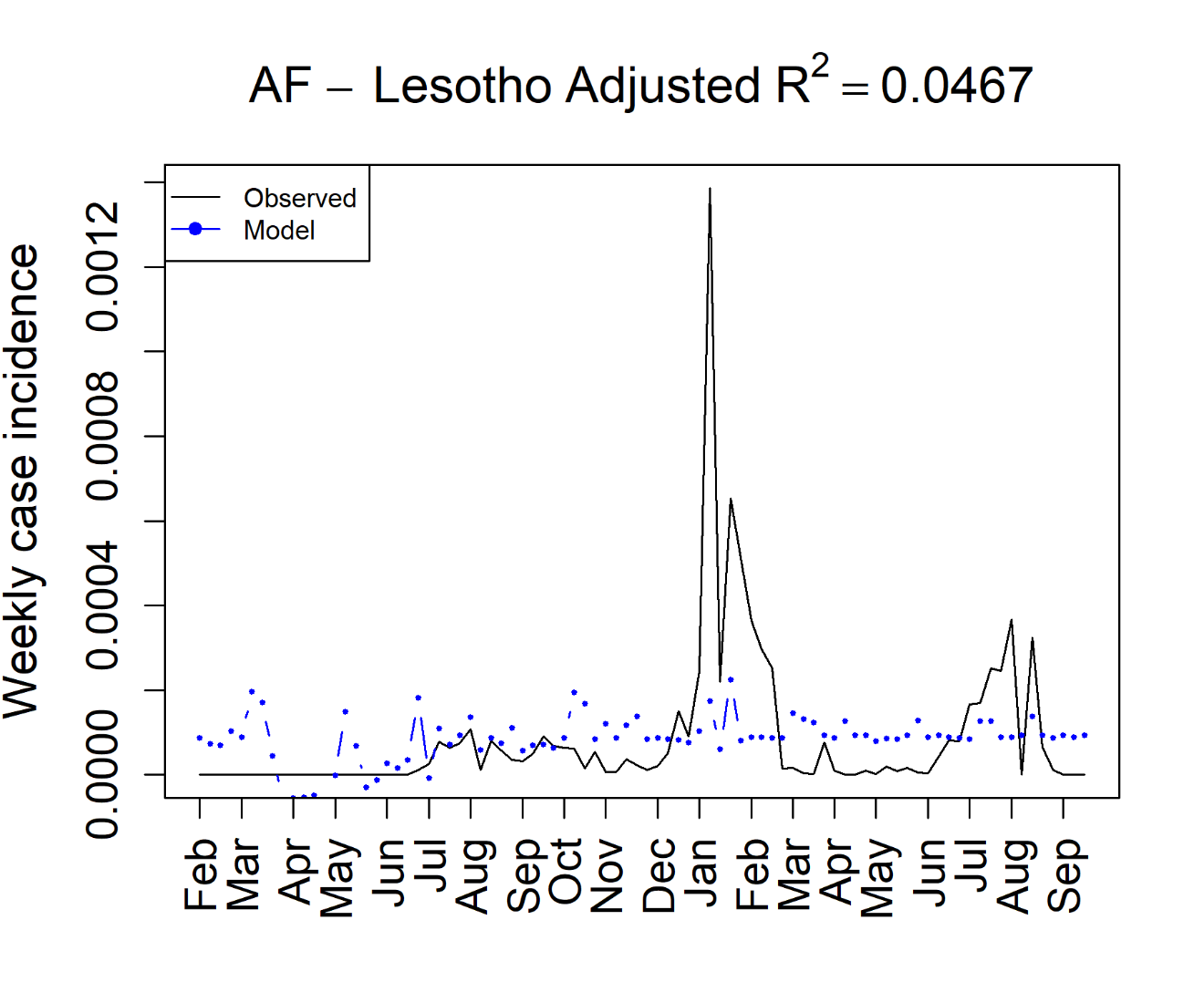

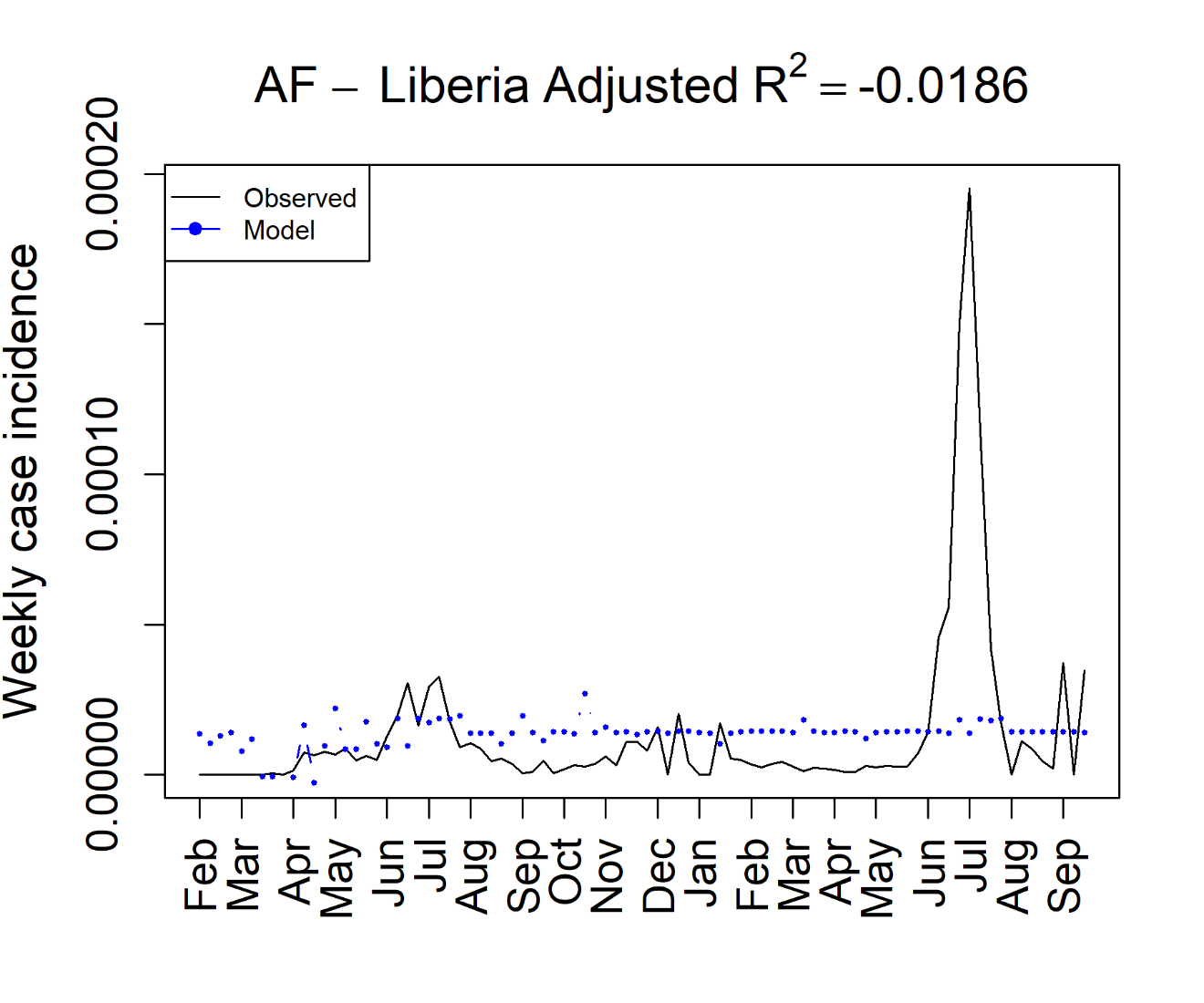

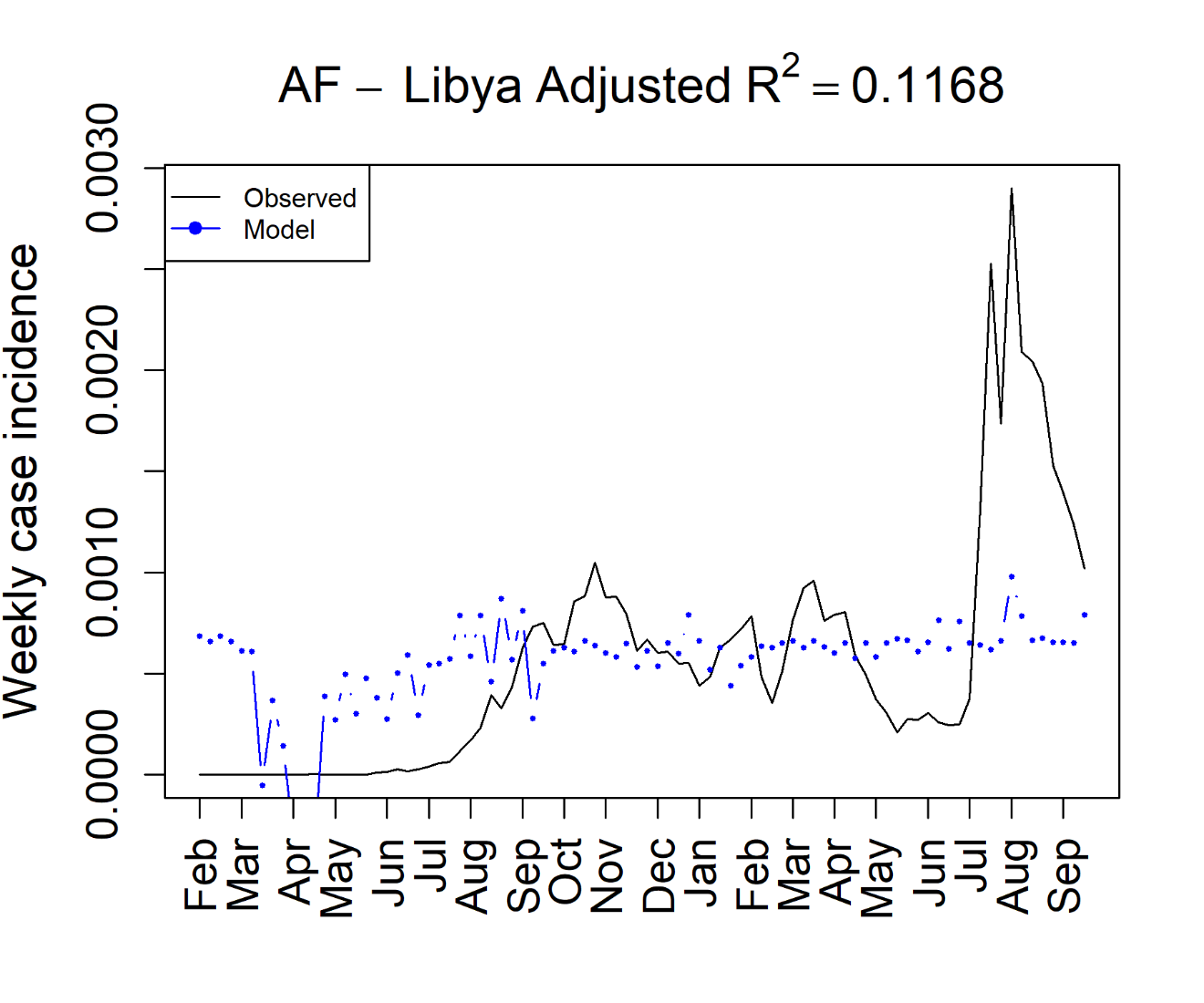

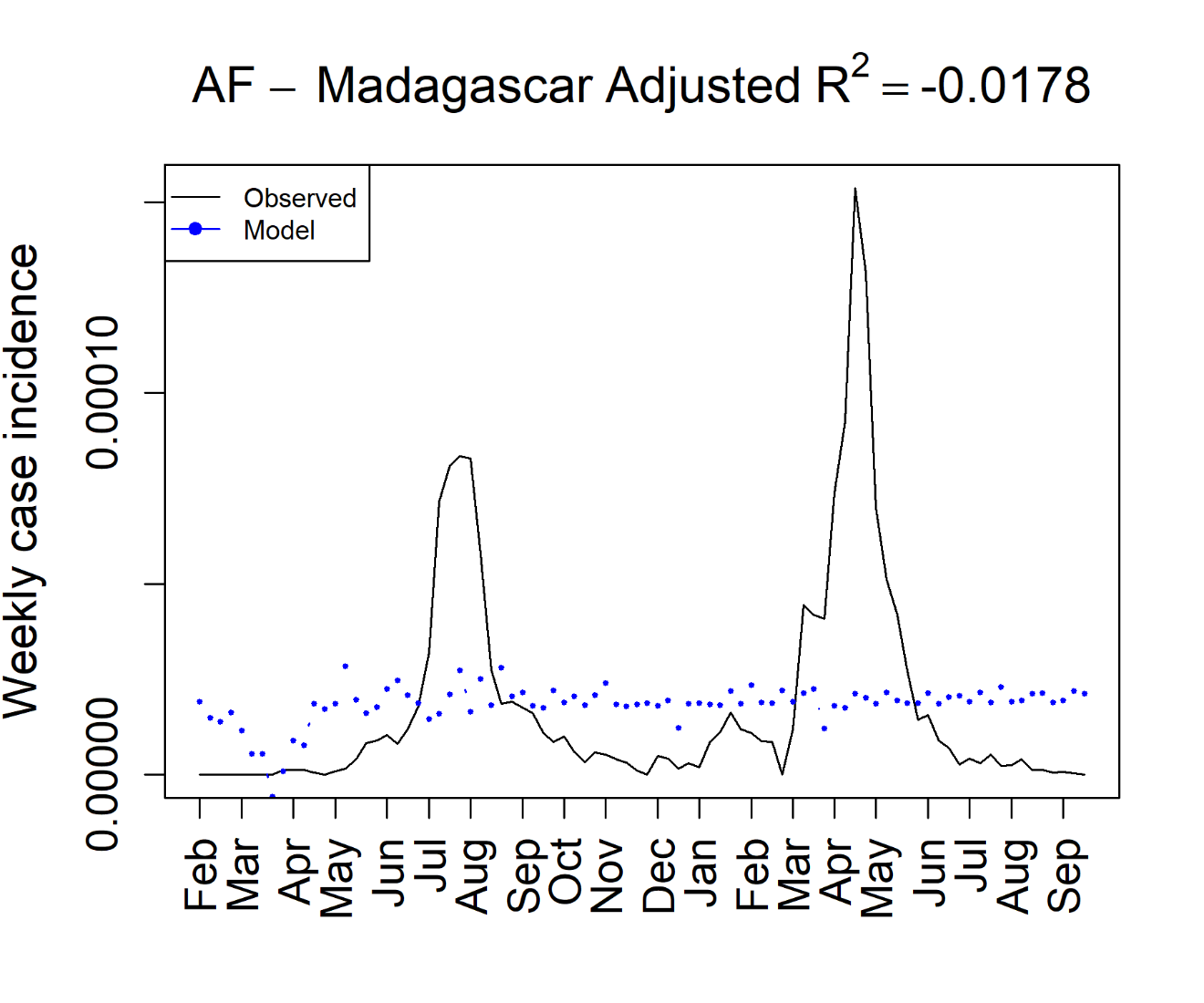

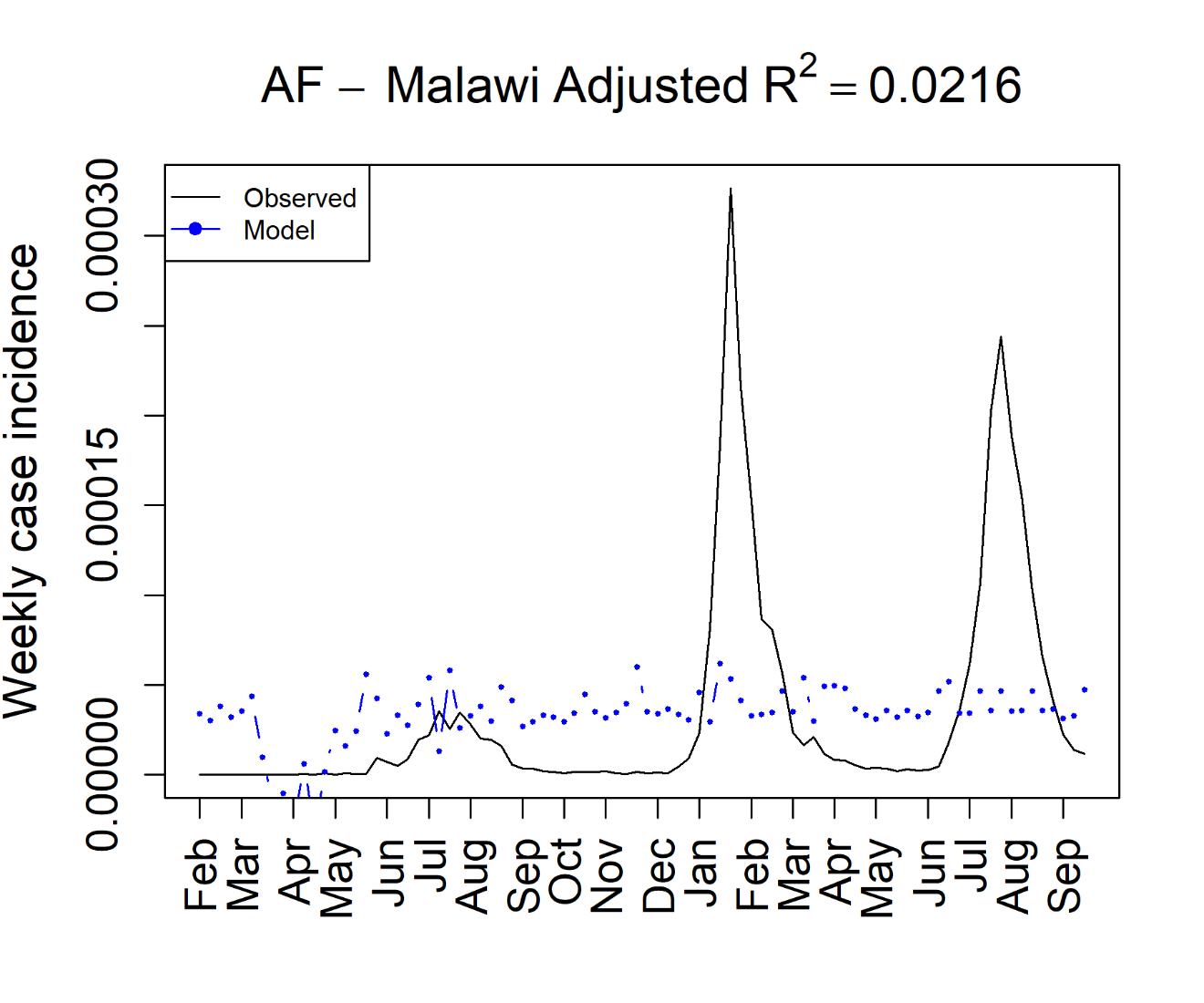

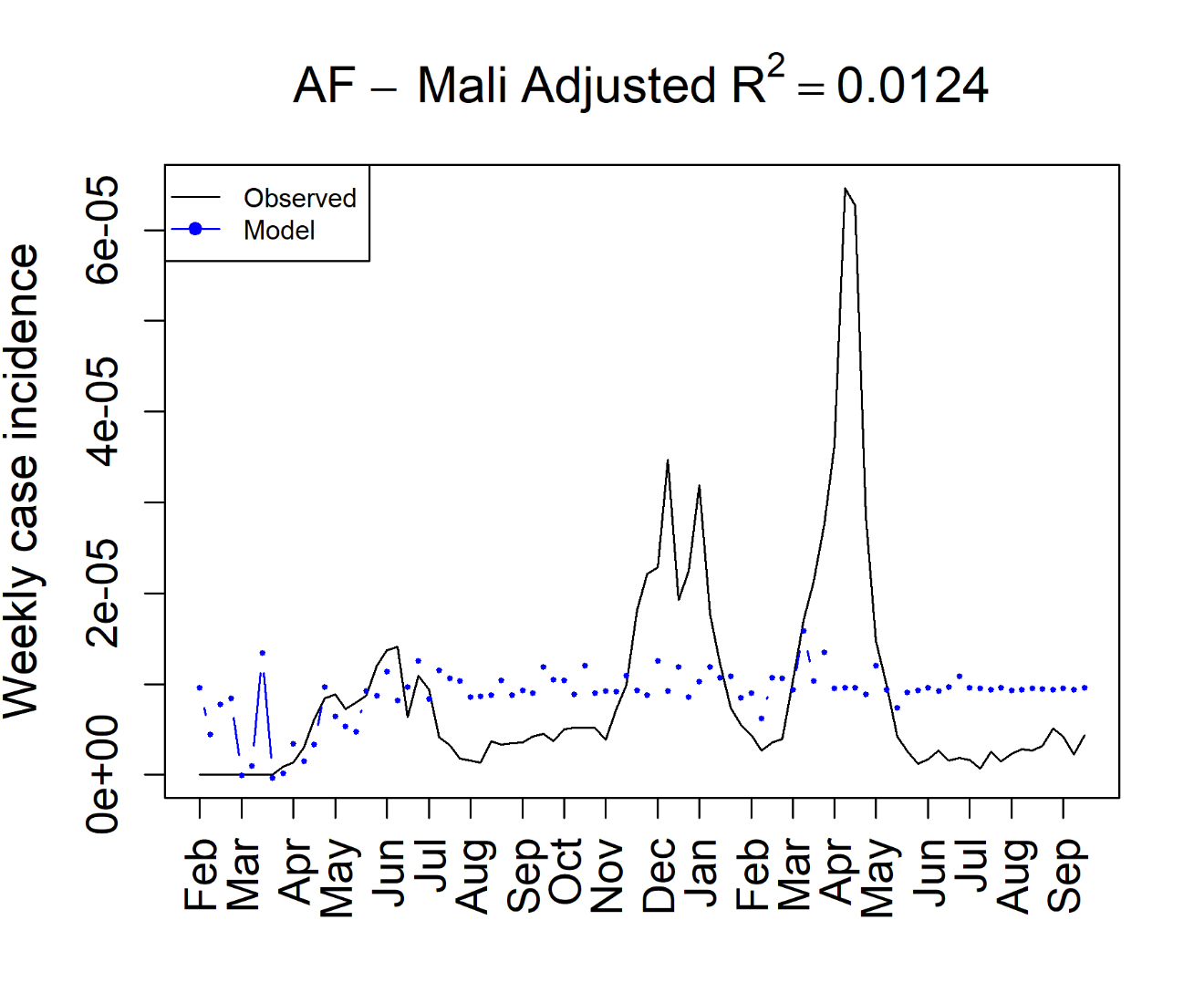

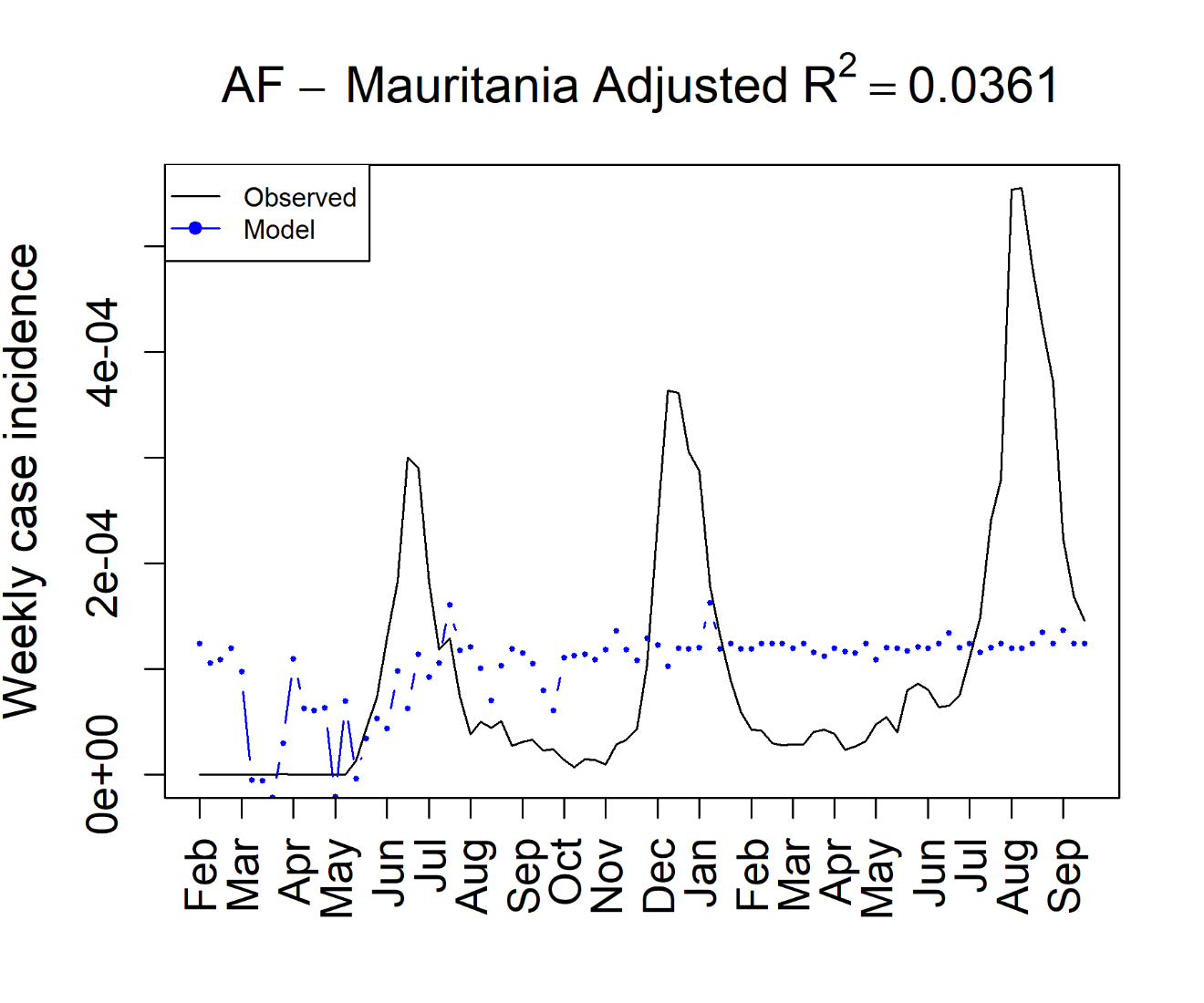

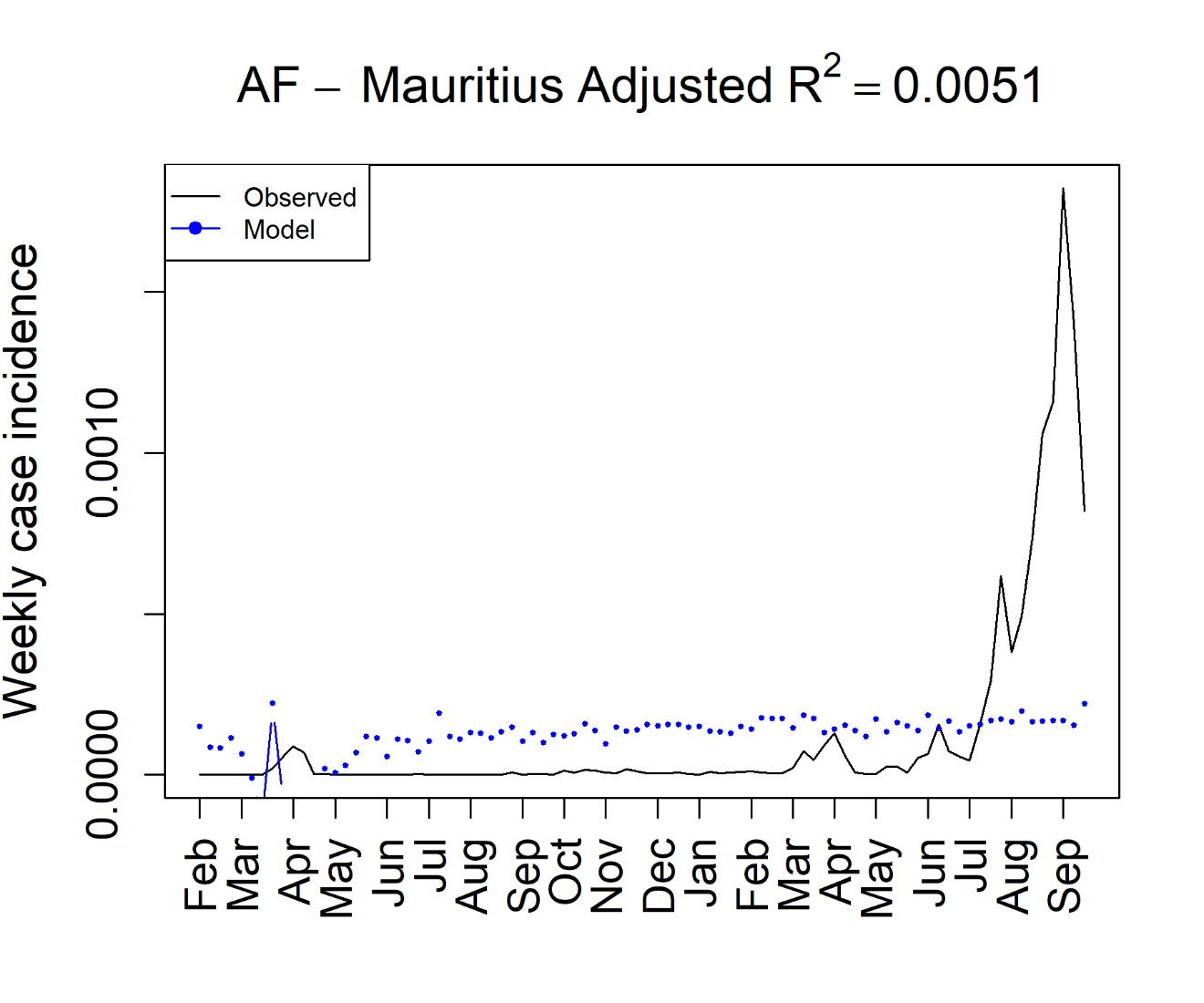

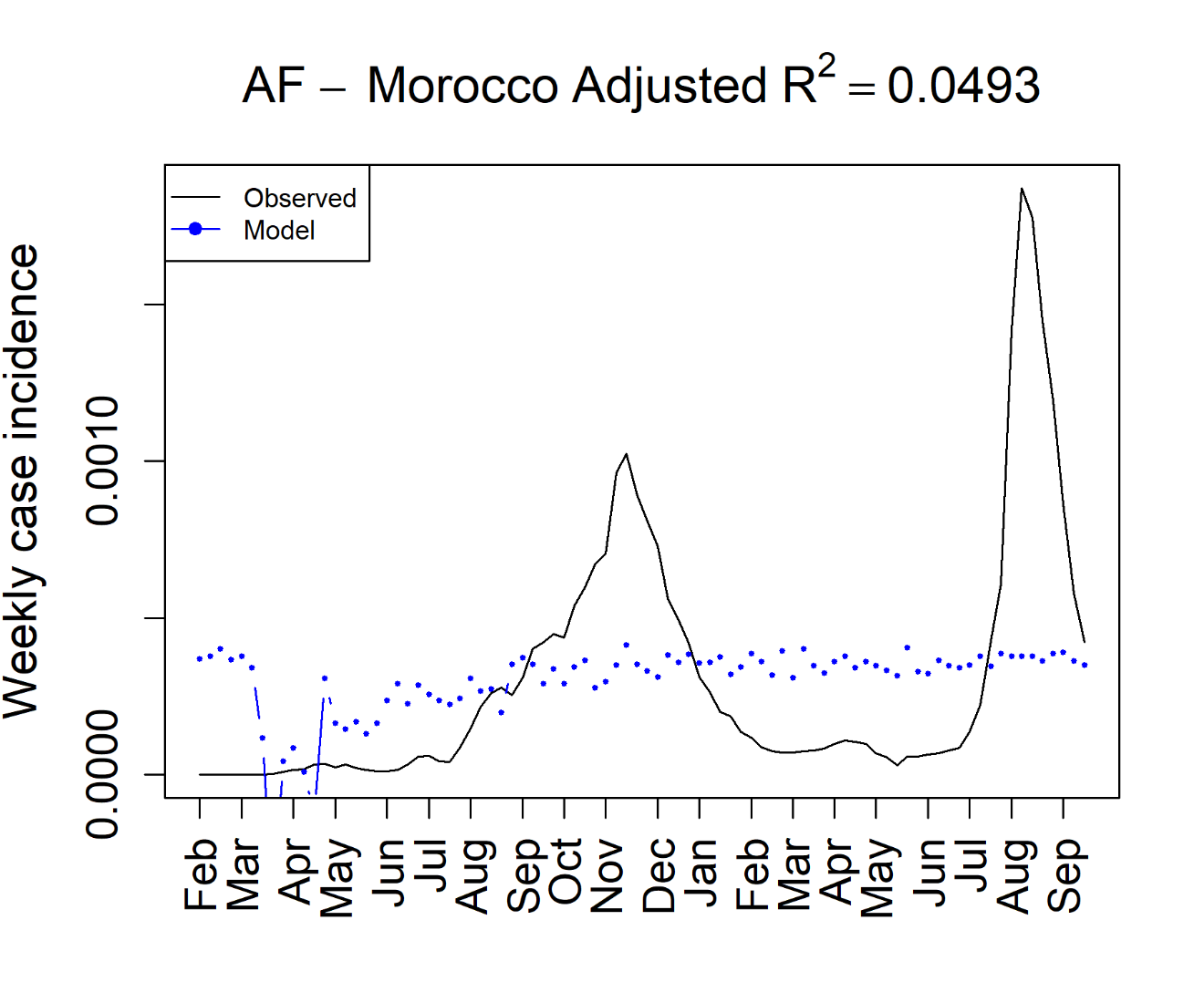

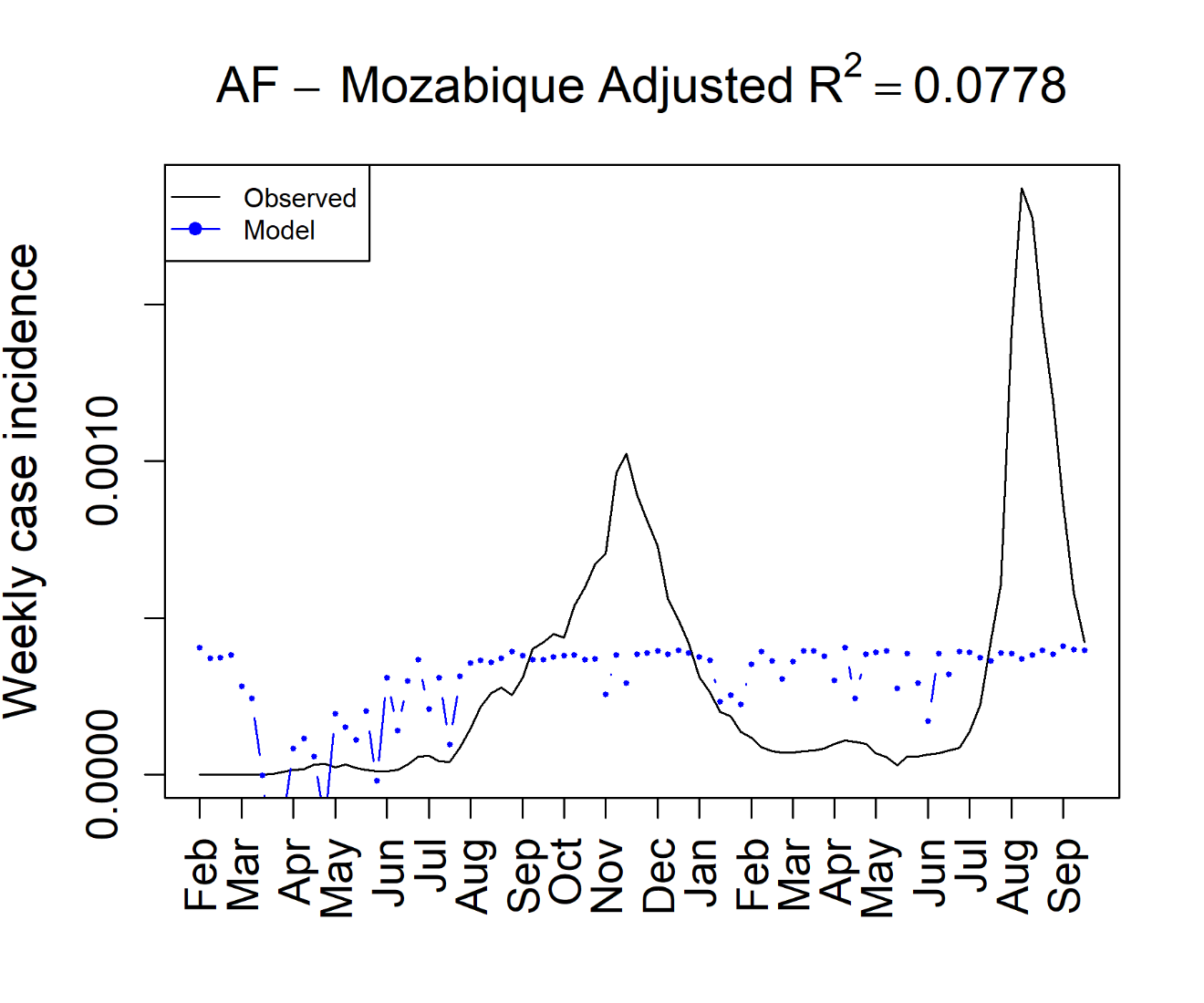

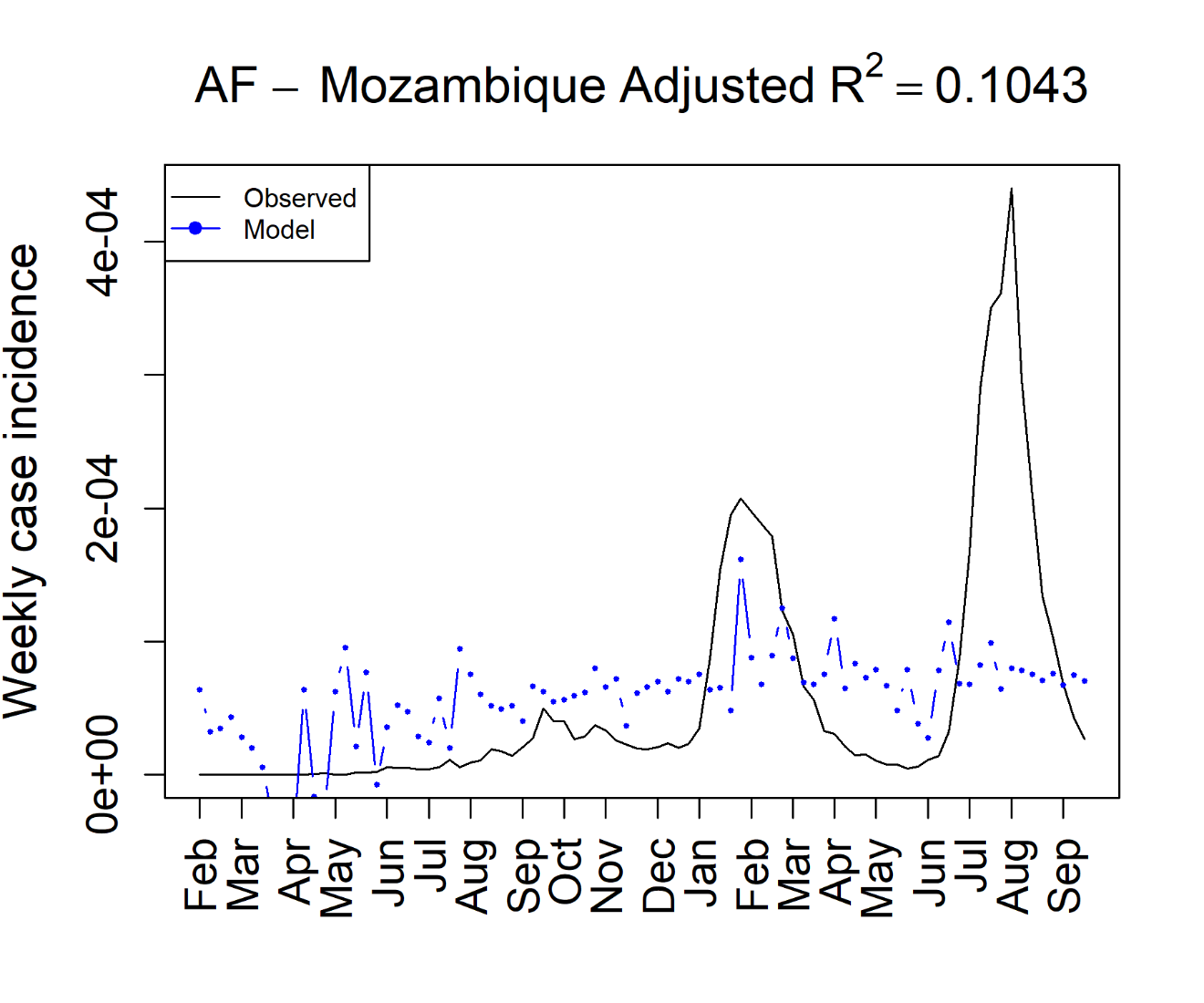

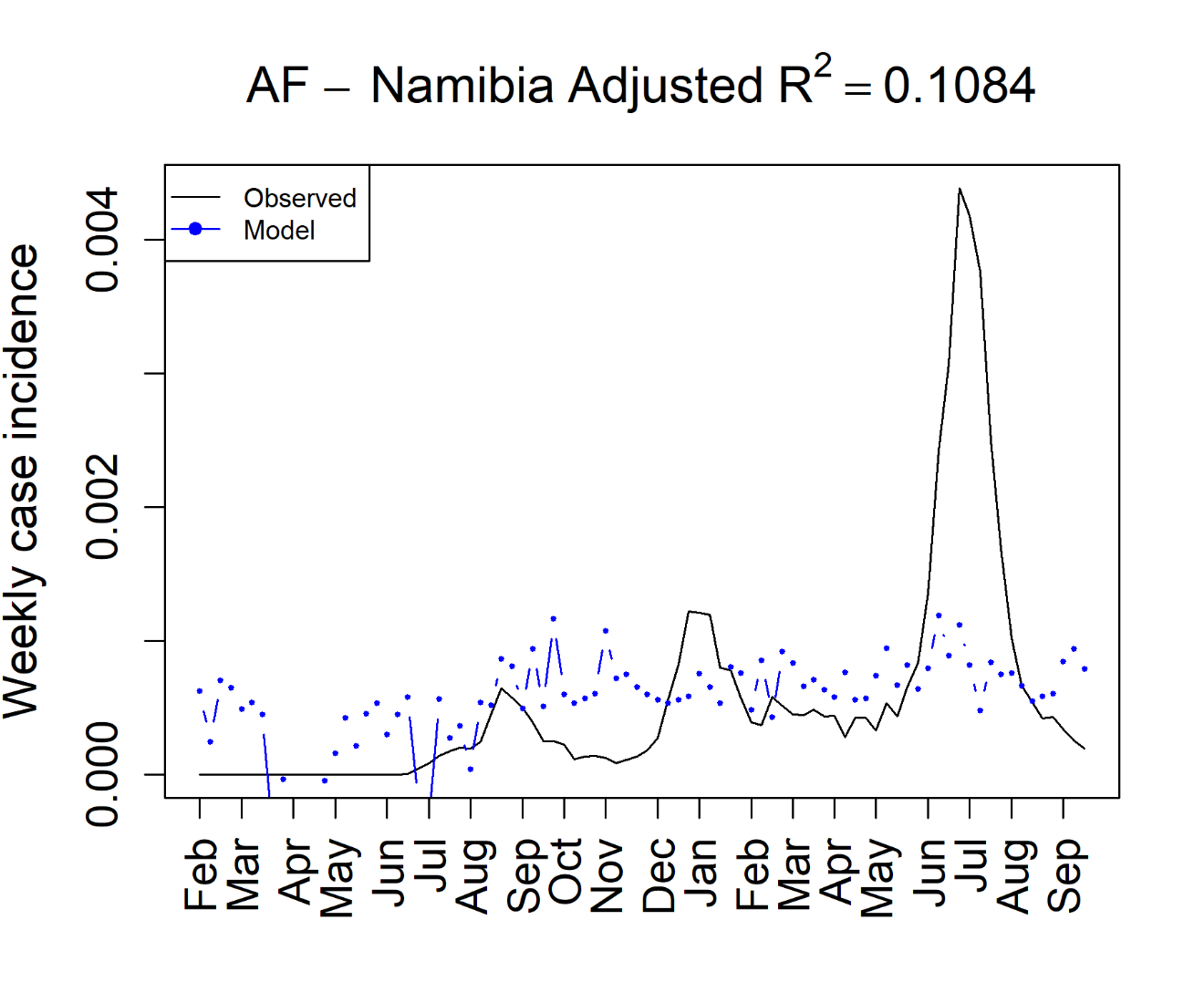

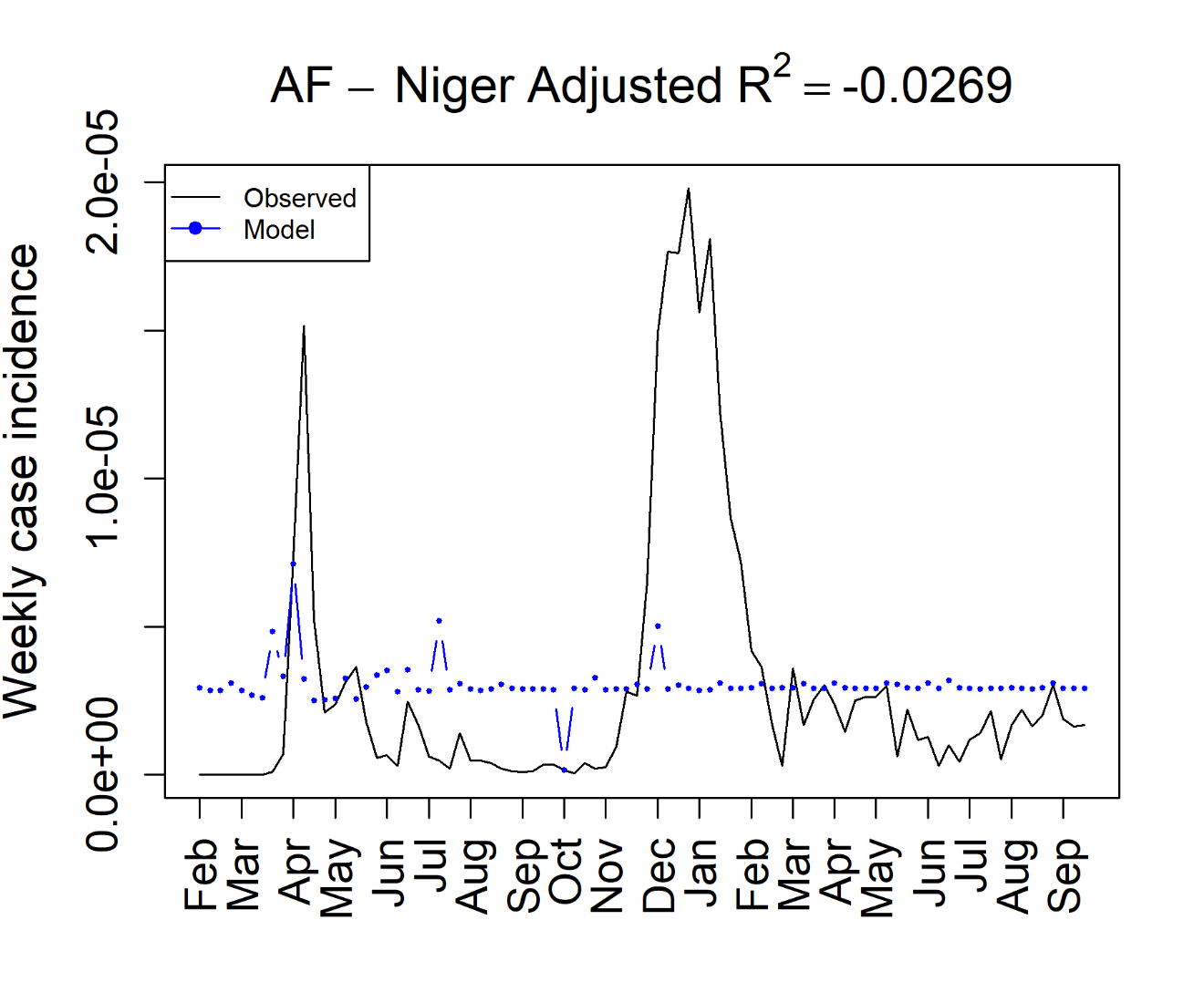

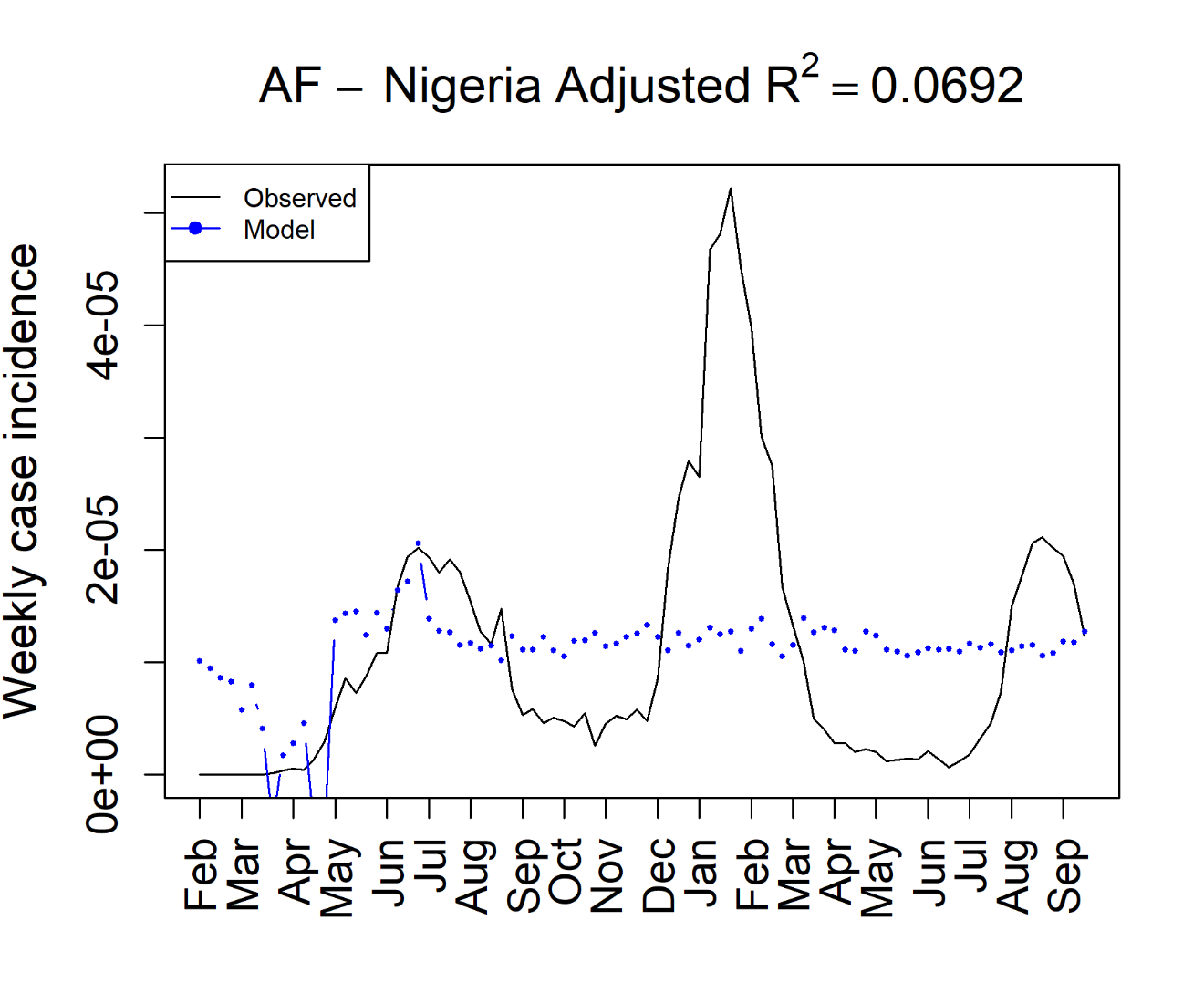

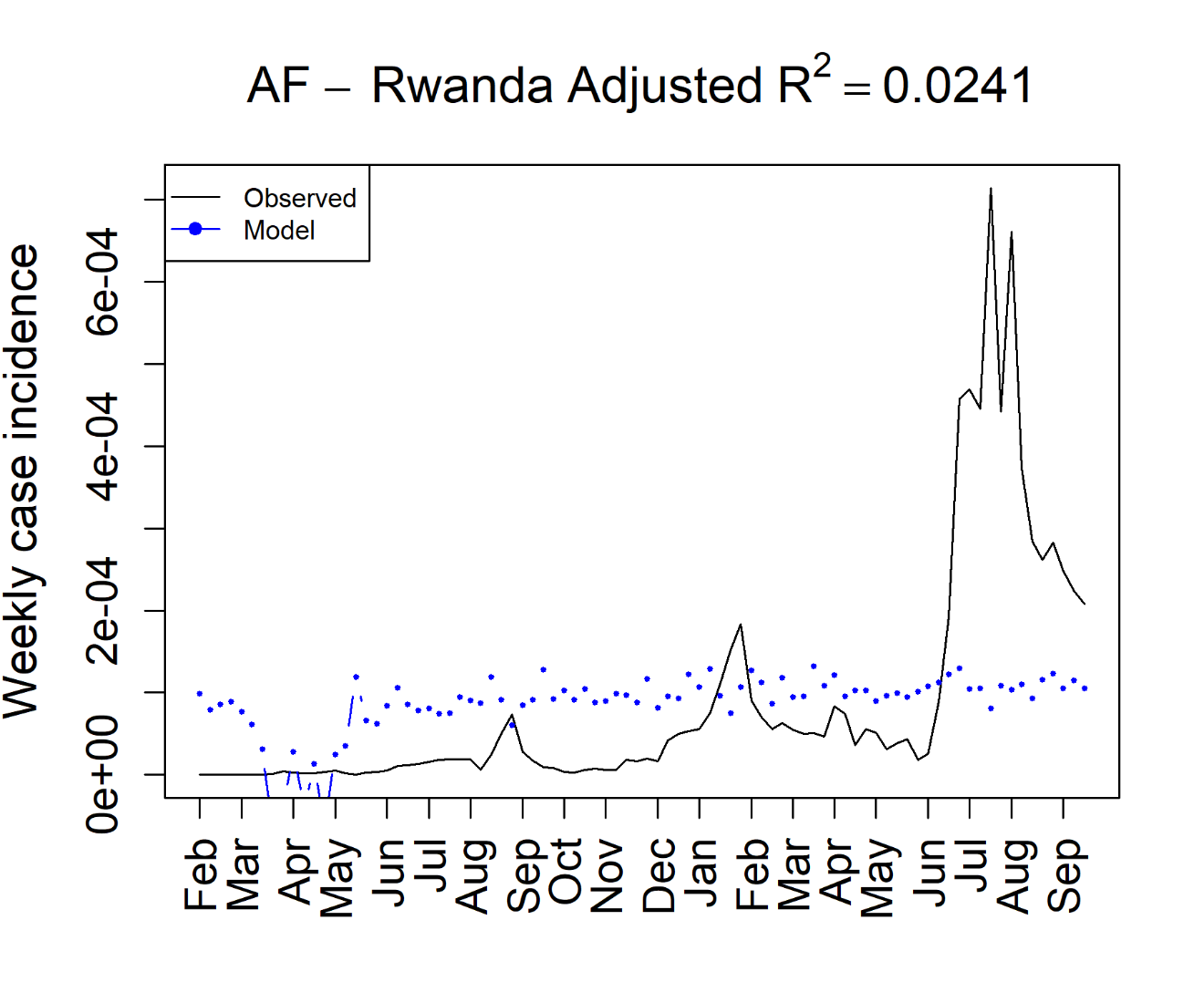

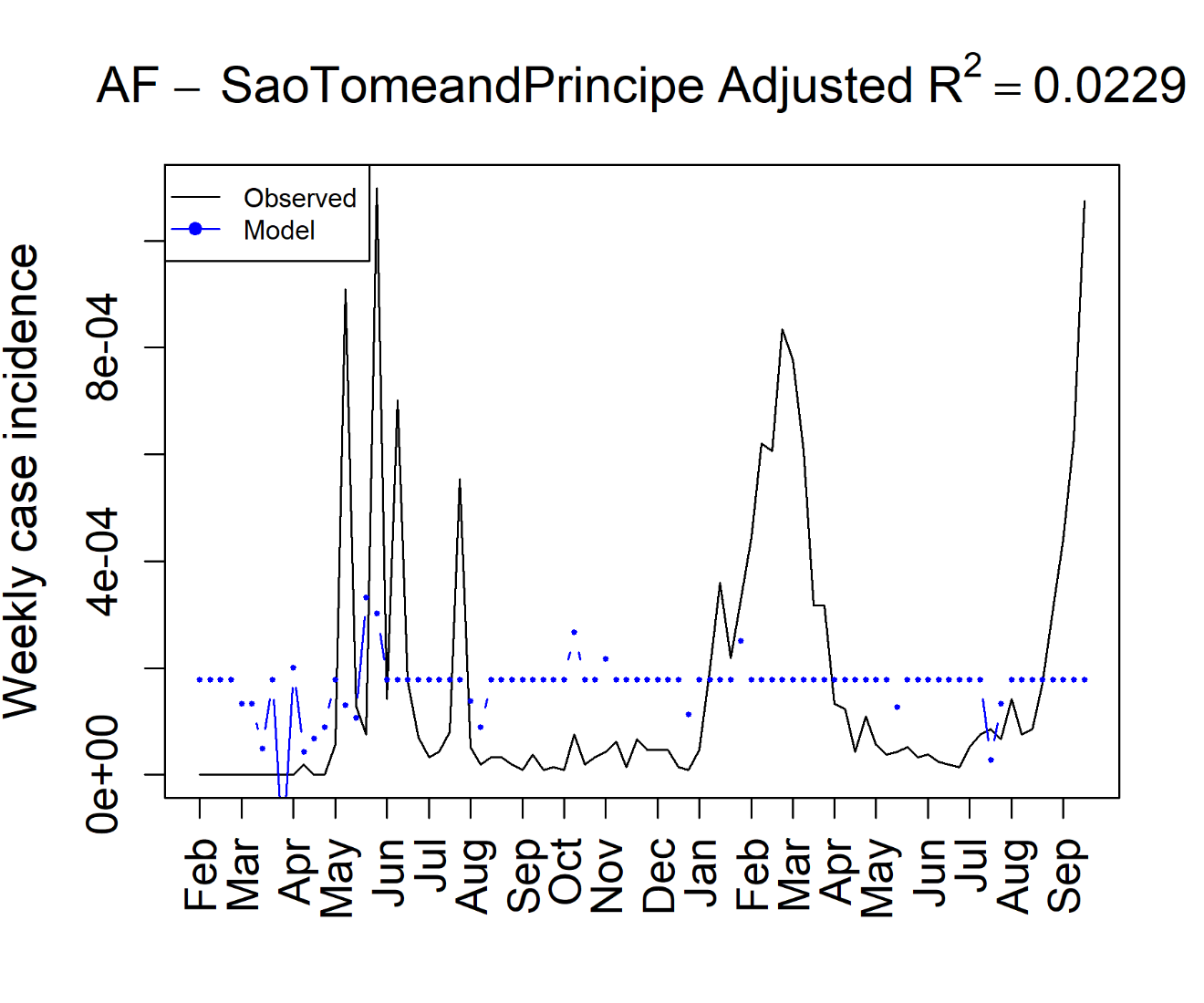

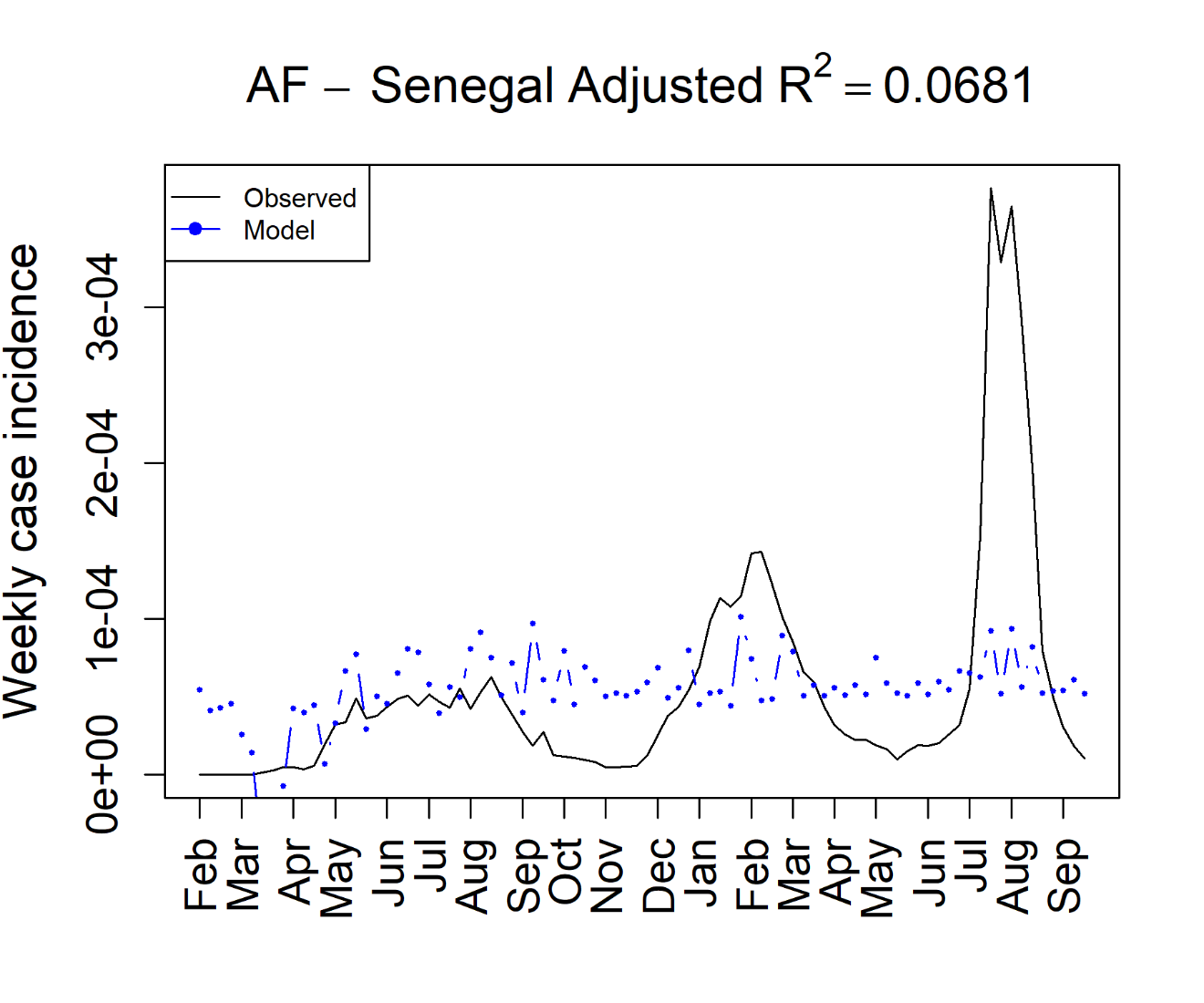

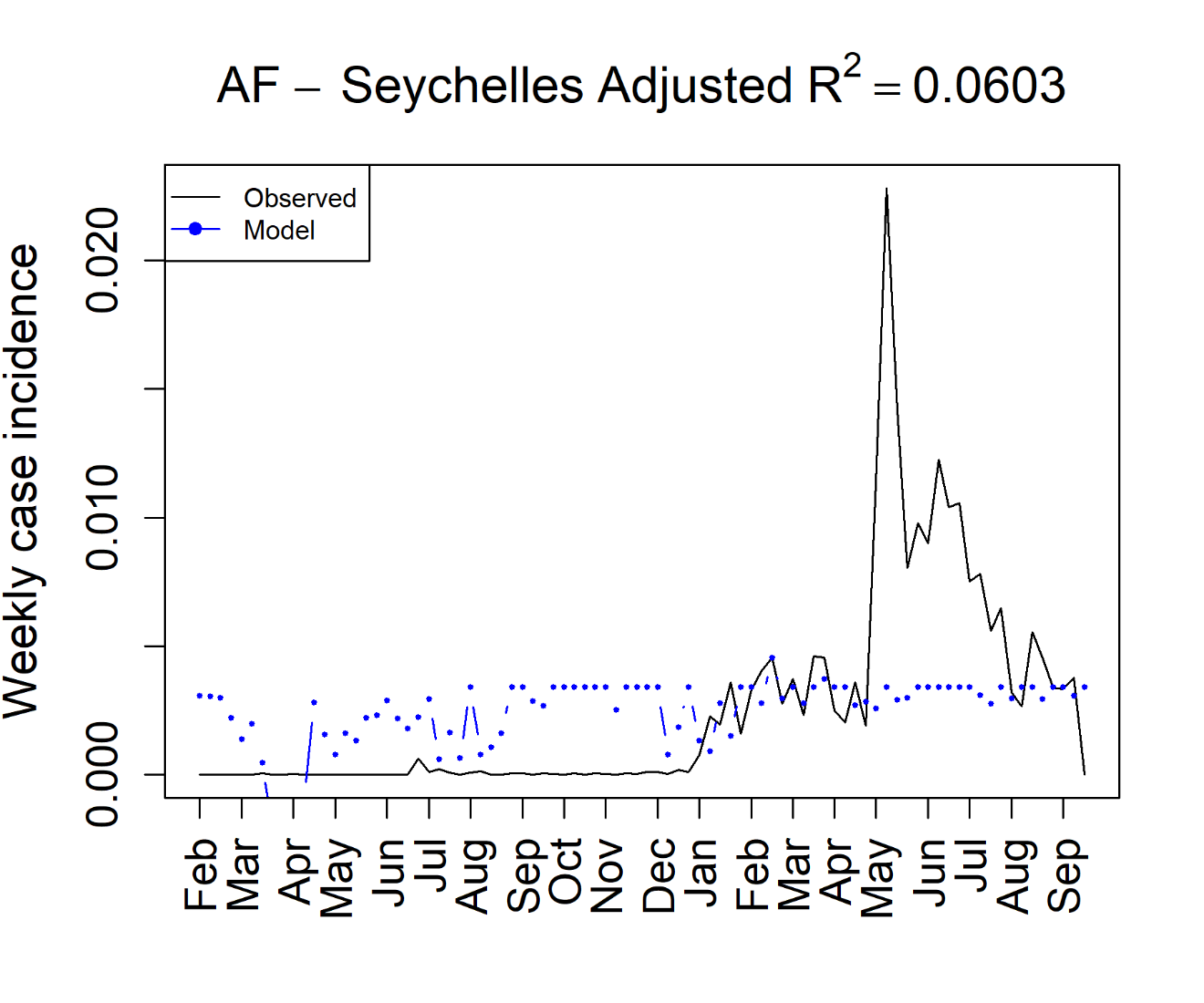

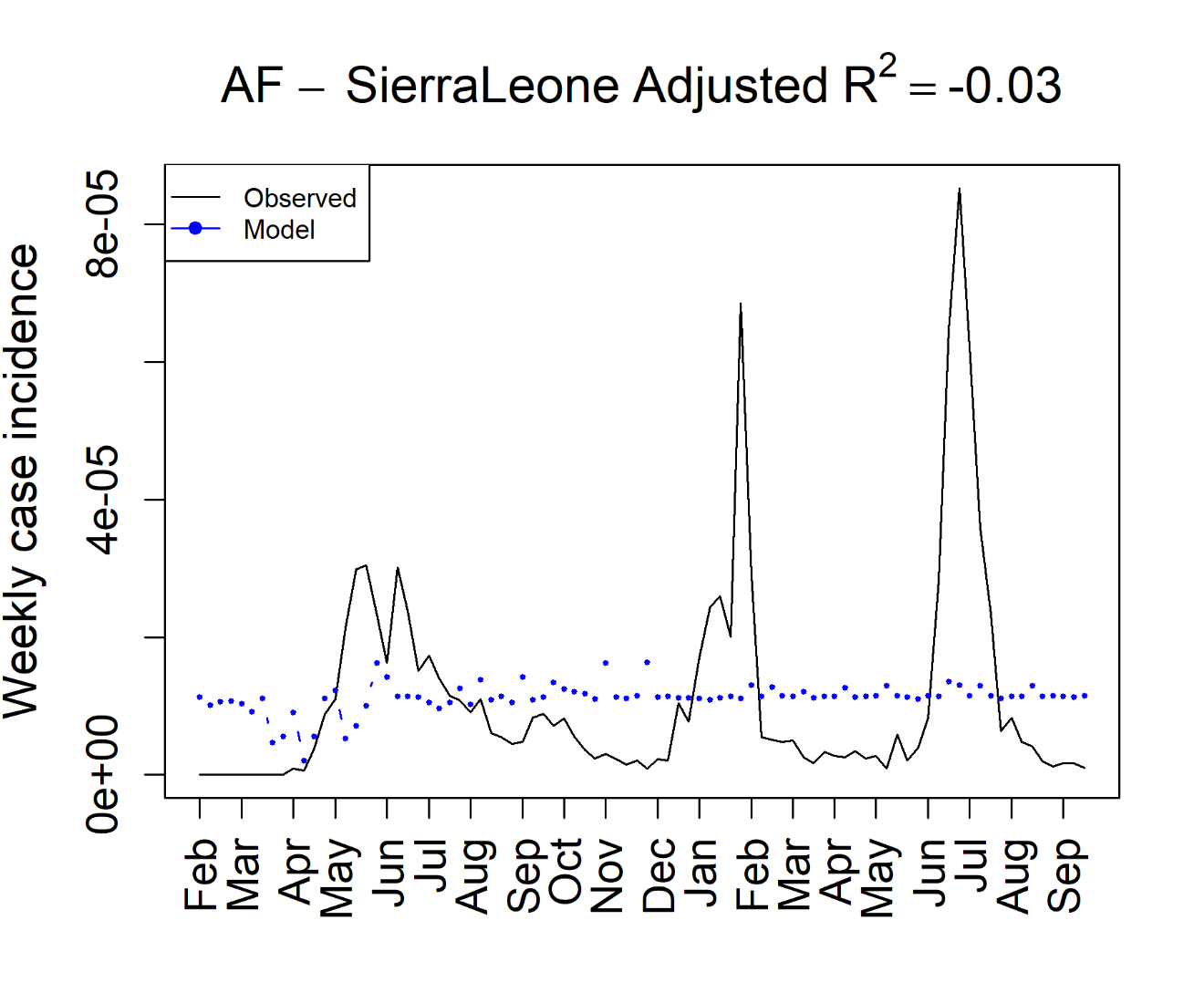

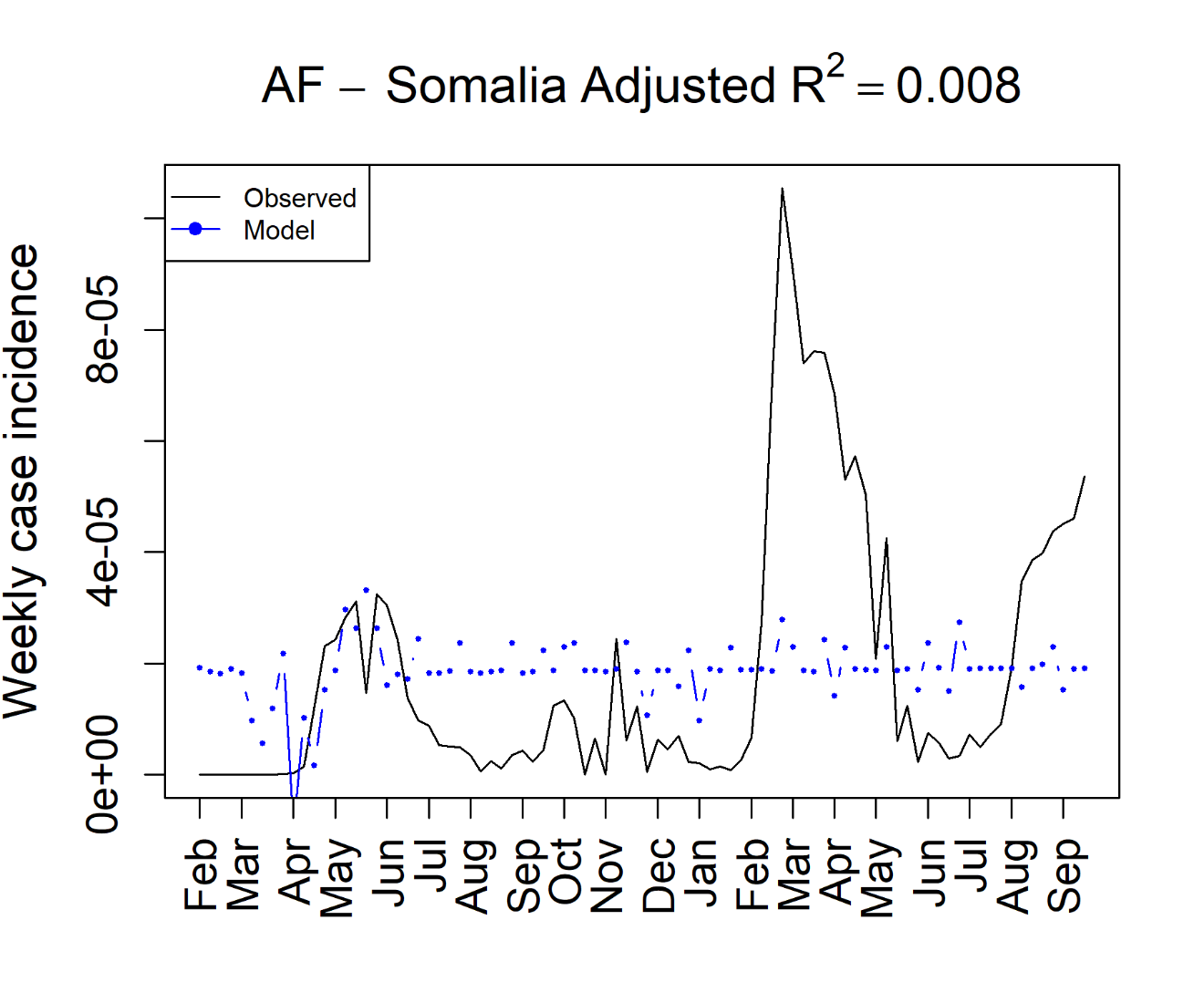

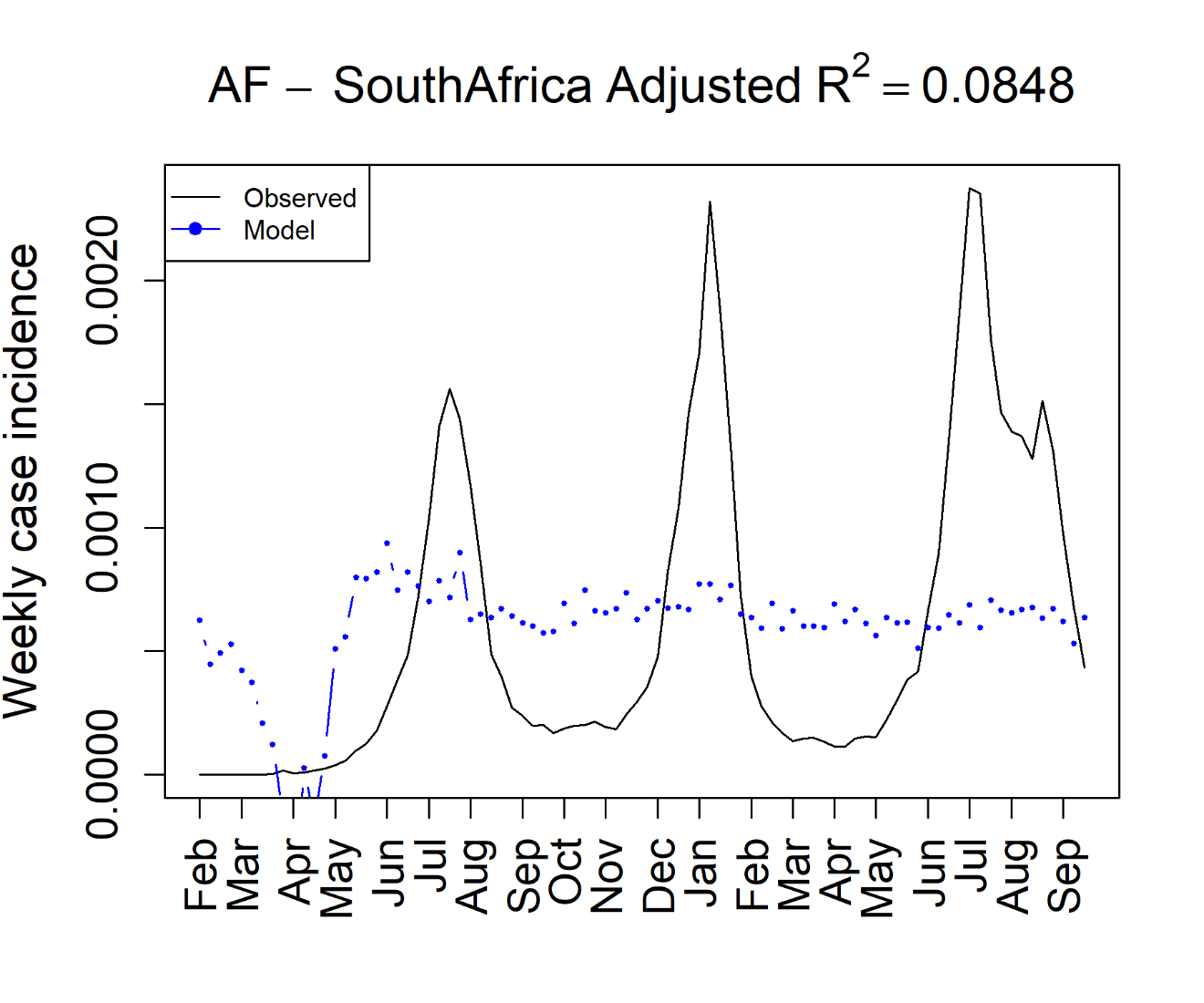

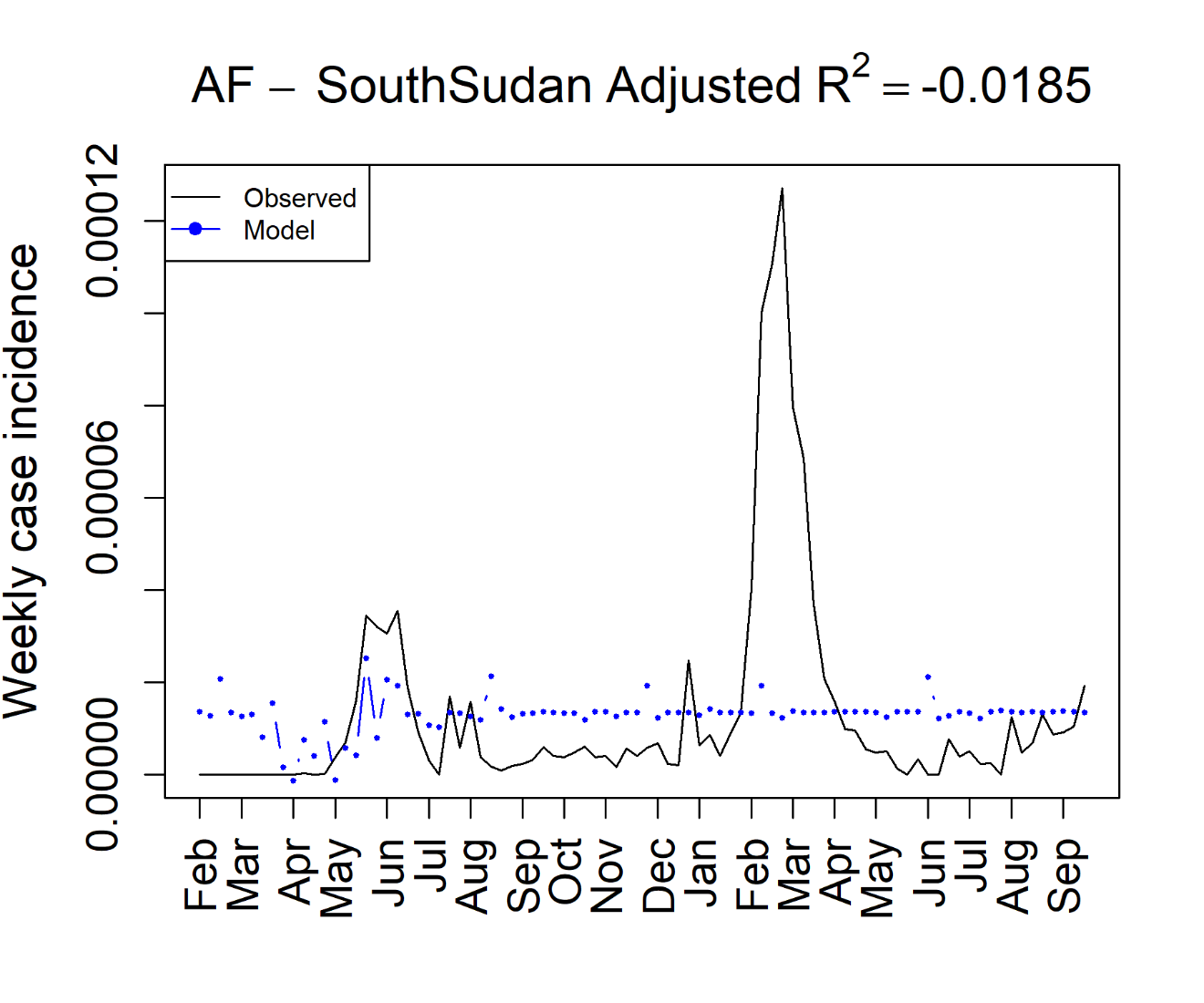

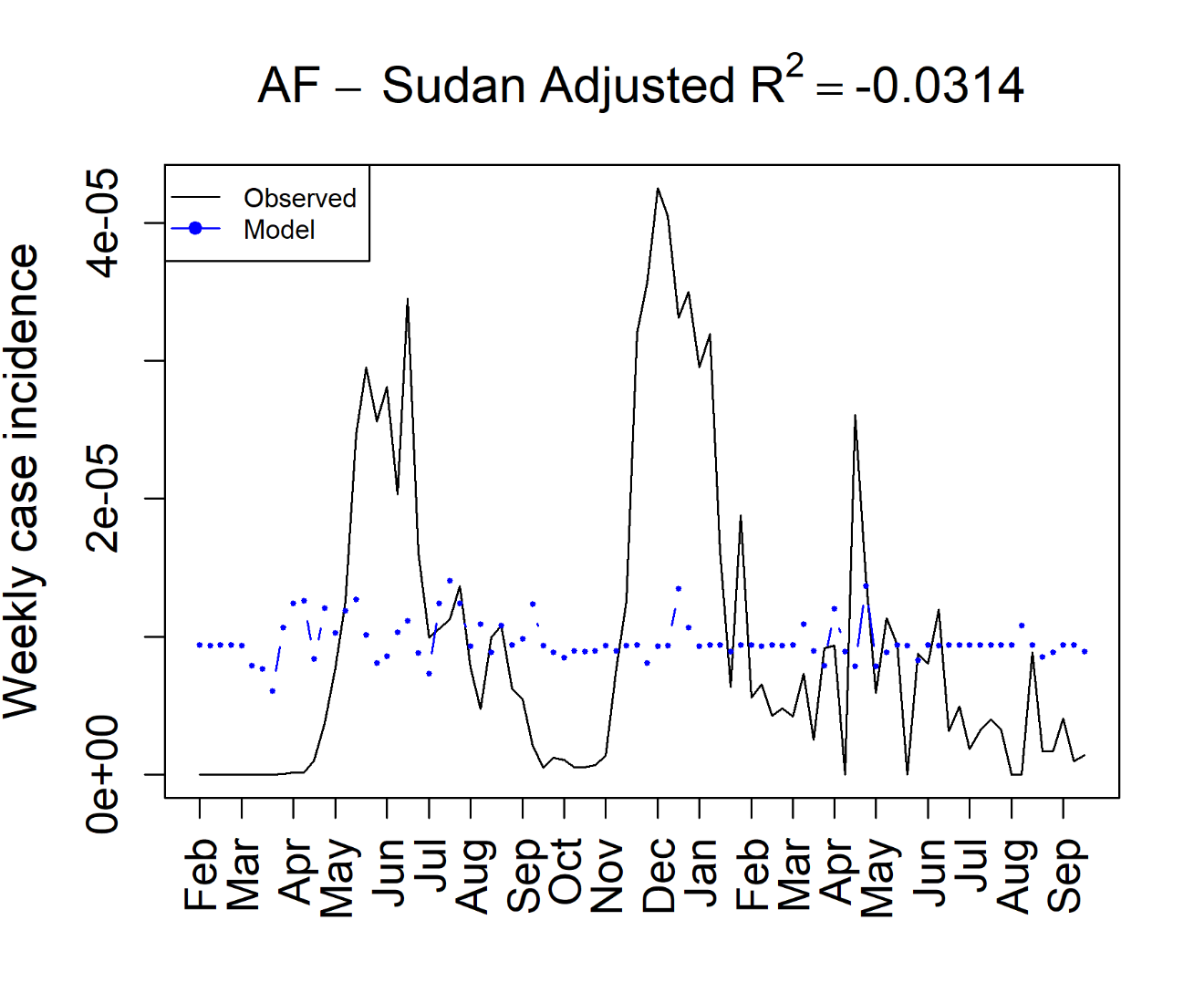

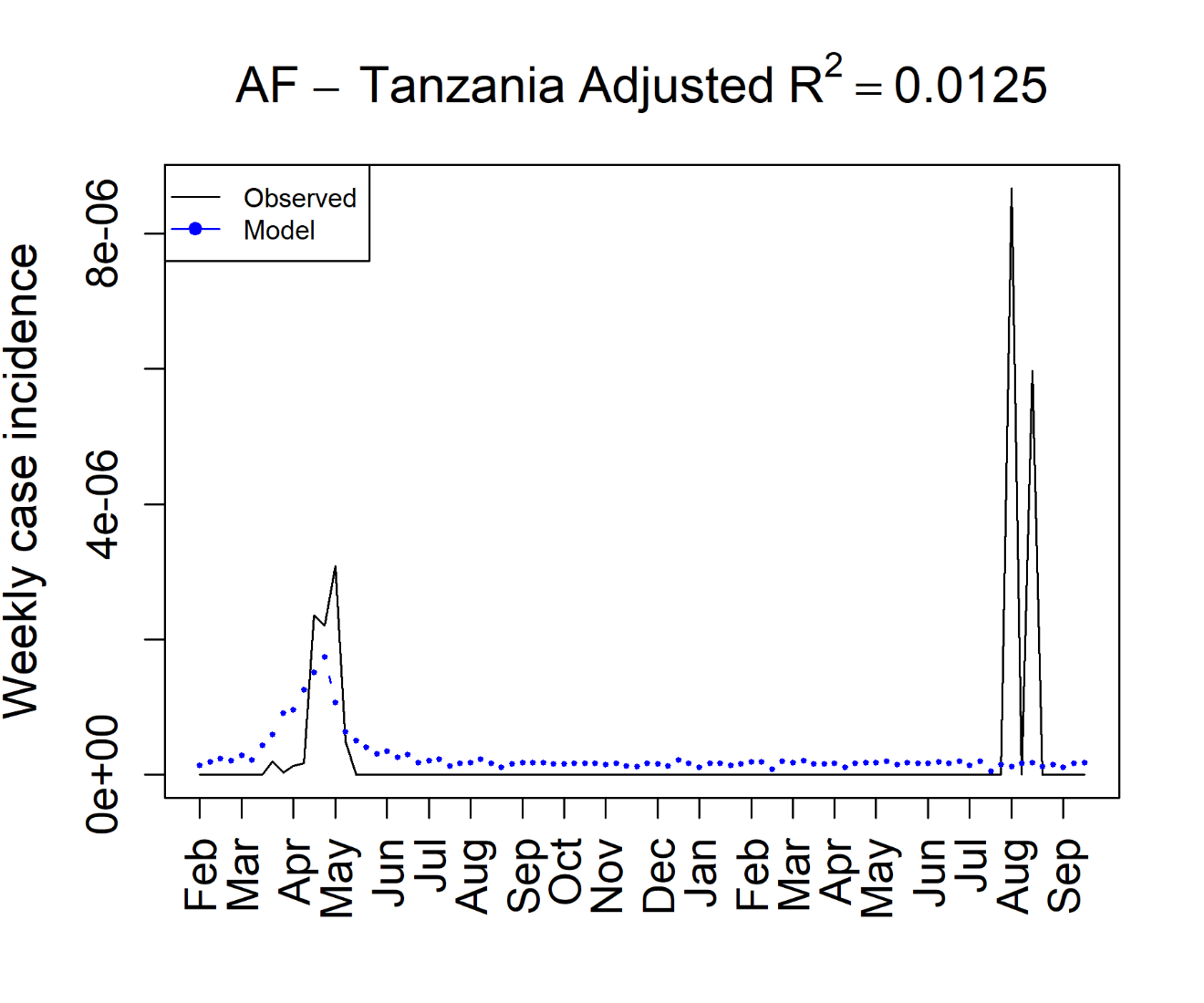

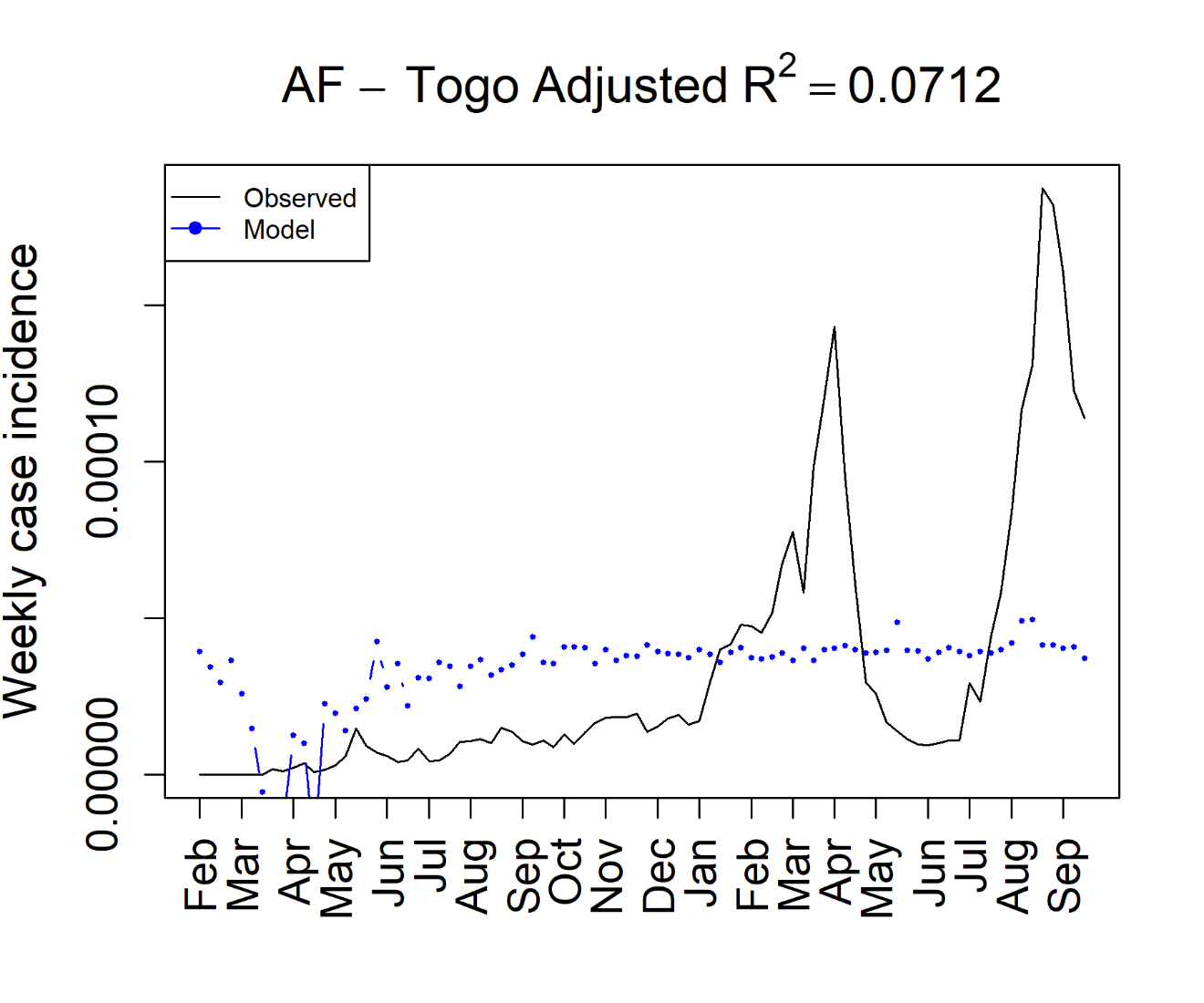

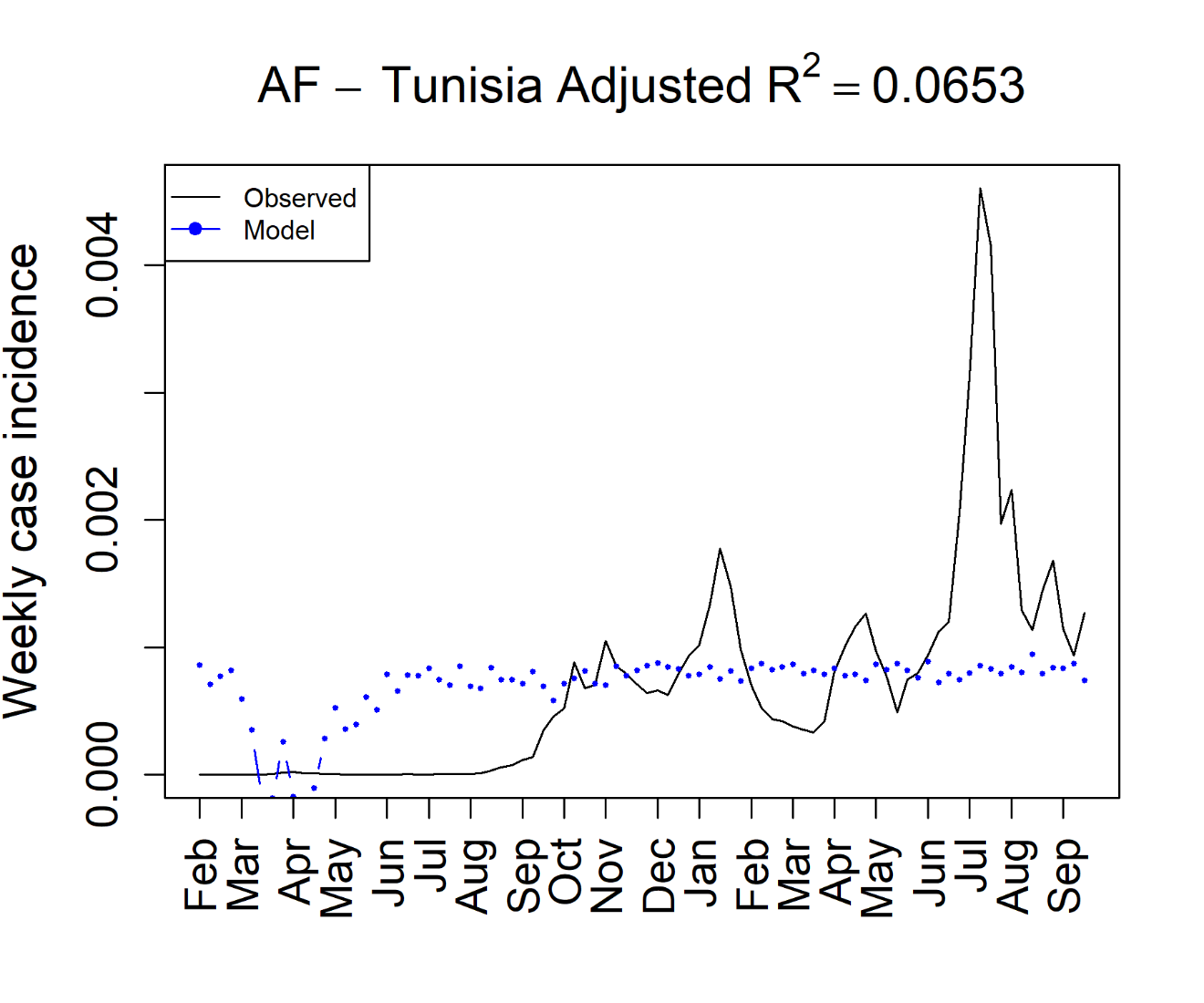

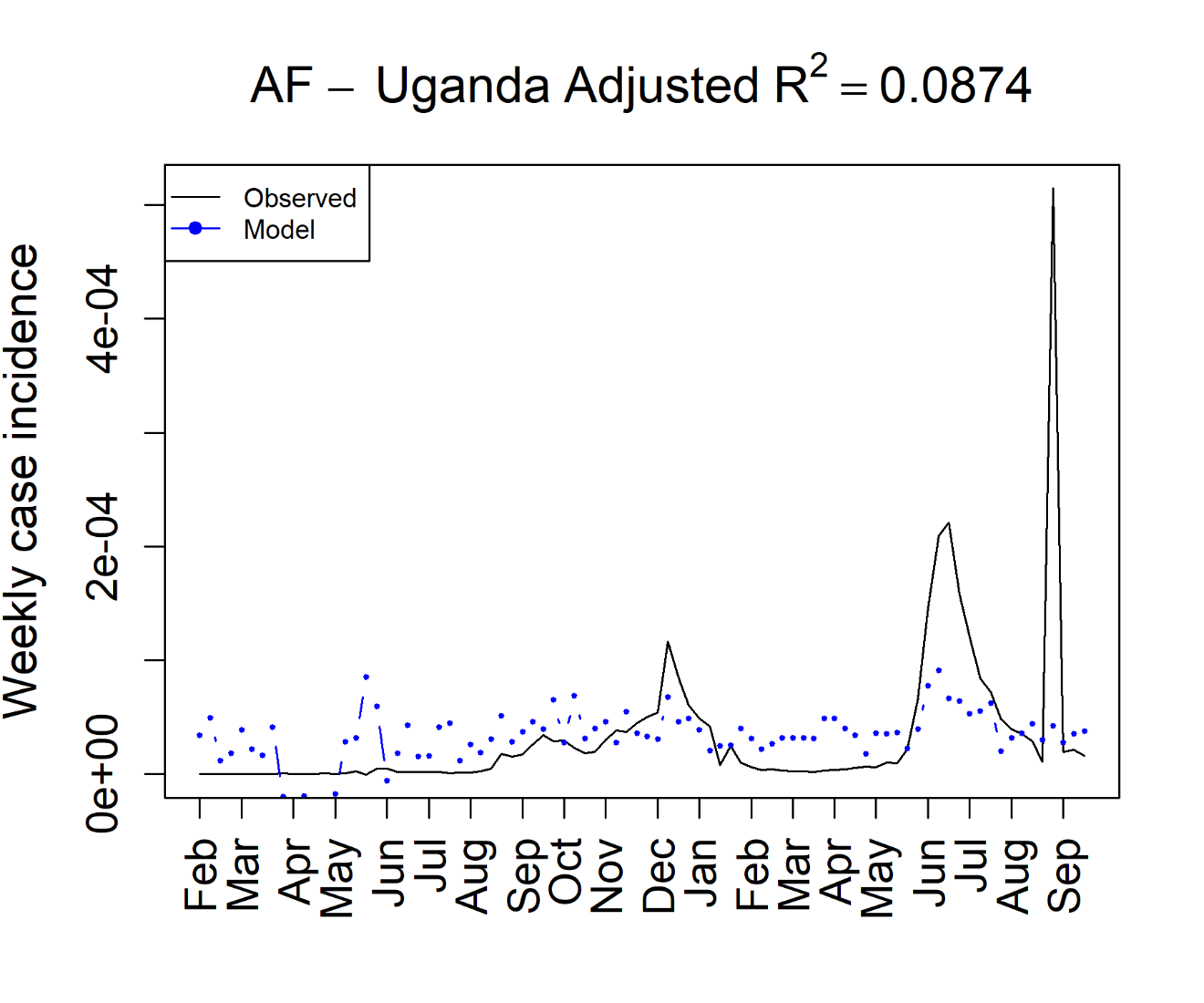

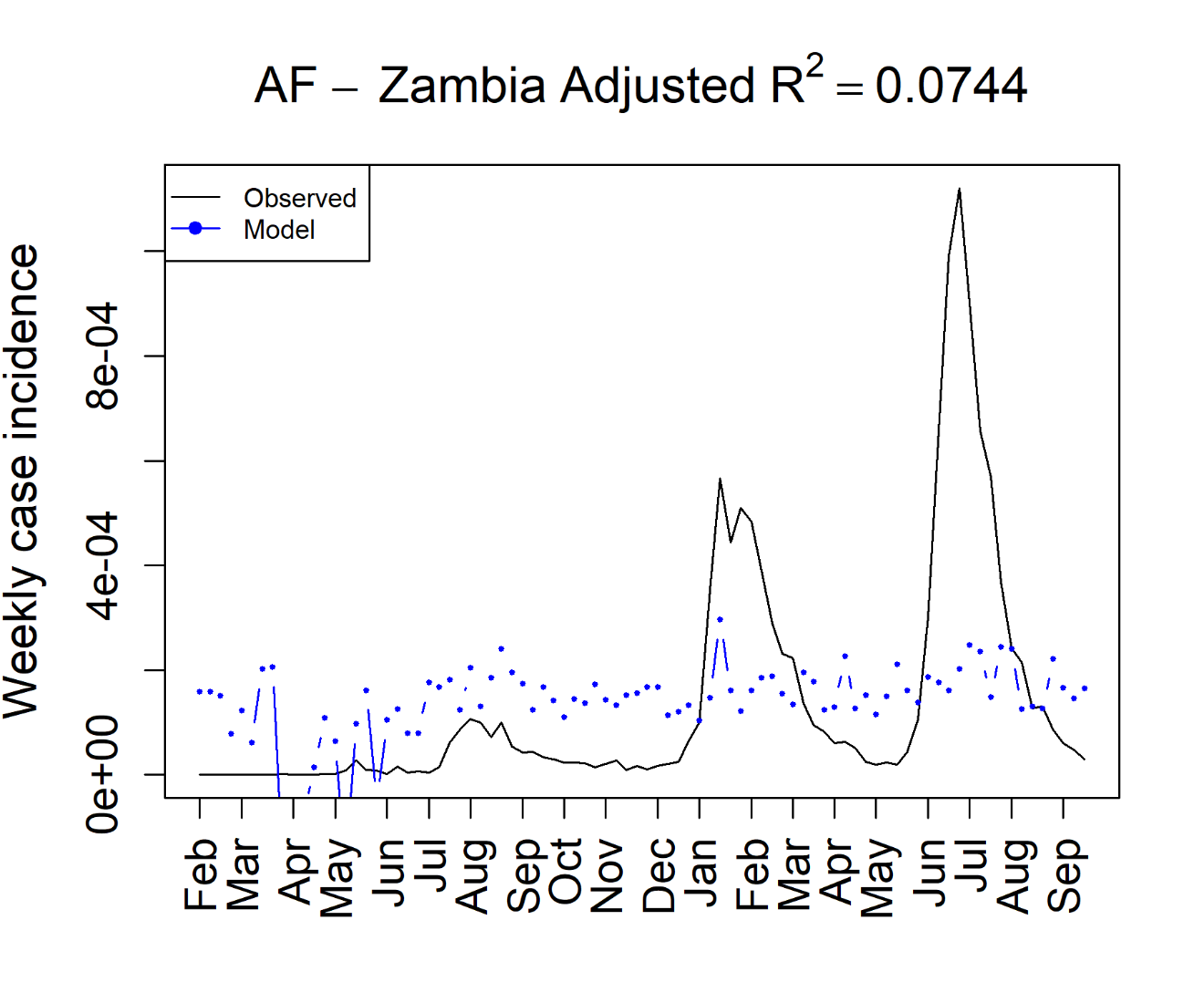

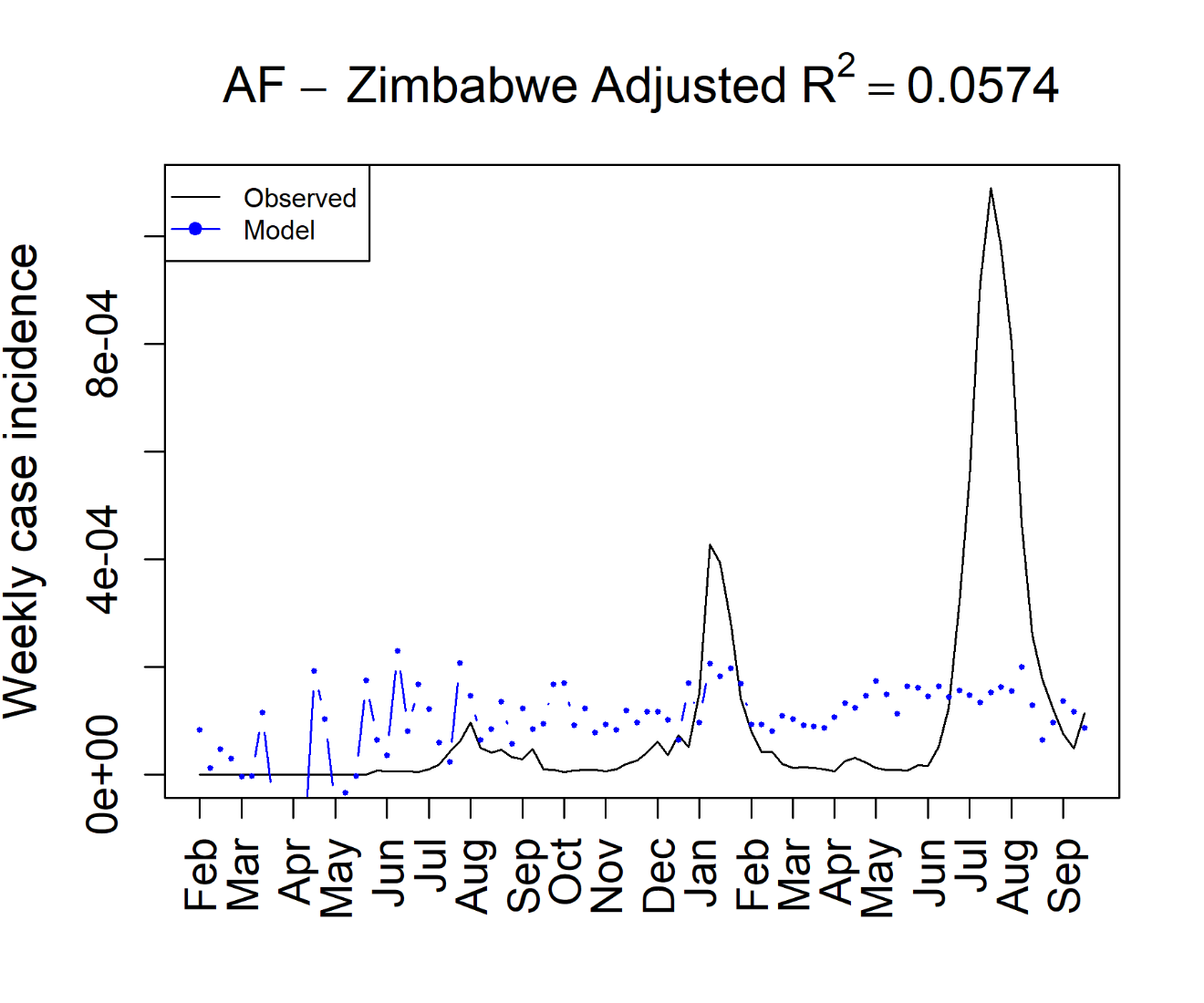

Supplement: S1 Fig — (DOCX) [file pone.0269573.s001.docx]
